# Supplementary material for: Selective sweep probabilities in spatially expanding populations
Source: Nat Commun. 2026 Feb 11;17:2181. doi: 10.1038/s41467-026-69363-7 (PMC12960945; doi:10.1038/s41467-026-69363-7)
Supplement: Supplementary file 1 — Supplementary Information [file 41467_2026_69363_MOESM1_ESM.pdf]

Supporting Information for

Selective sweep probabilities in spatially expanding populations

Alexander Stein, Kate Bostock, Ramanarayanan Kizhuttil, Maciej Bak and Robert Noble

Robert Noble.
E-mail: robert.noble@citystgeorges.ac.uk

This PDF file includes:

Supporting text
Figs. S1 to S17
Tables S1 to S3
SI References

Supporting Information Text

1. Proof of Claim 1

Proof of claim 1. By definition of the per capita mutation rate, we have $\mu N(s)ds$ successful mutations in the infinitesimal time interval $[s, s+ds]$. We sum over the time interval $[0,t]$ and obtain $\lambda = \int_0^t \mu N(s)\,ds$. We assume that mutations occur independently of each other (infinite alleles) such that the stochastic process is a non-homogeneous Poisson process which has a Poisson distribution with mean λ. □

2. Connection between per-capita and per-division mutation rate

In our macroscopic model, we assume a constant per-capita mutation rate $\tilde{\mu}$. Often, it is assumed that mutations are coupled to division. Here, we show how a per-capita mutation rate connects to a per-division mutation rate. Suppose that after each division one of the two daughter cells obtains a driver mutation with probability p_μ. (The probability that both daughter cells obtain a driver mutation scales with p_μ^2, which can be neglected for sufficiently small p_μ.) Suppose cells divide at rate b and die at rate d. If division rates are constant over time and space, then the per-division mutation probability p_μ yields a constant per-capita mutation rate $\tilde{\mu} = p_\mu b$. The death rate may still differ in time and space.

3. Exact solution for the conditional sweep probability

We compute the full exact expression for the conditional sweep probability $\Pr(\text{sweep}|X = x, Y = y)$ in 3D (eqn. 9 in the main text). From the main text, we have

$$\Pr(\text{sweep}|X = x, Y = y) = e^{-\mu \int_0^\infty N_{\text{wt}}(\tau)\,d\tau}. \quad [1]$$

were $N_{\text{wt}}(\tau)$ is the size of the remaining wildtype population given in eqn. 6 in the main text. The integral is divided into

$$\int_0^\infty N_{\text{wt}}(\tau)\,d\tau = \int_0^{\tau_1} \tilde{N}_{\text{wt}}(\tau) - \Delta_1(\tau)\,d\tau + \int_{\tau_1}^{\tau_2} \tilde{N}_{\text{wt}}(\tau) - \Delta_2(\tau)\,d\tau. \quad [2]$$

Here, $\tilde{N}_{\text{wt}}(\tau)$ and $\Delta_1(\tau)$ are the population sizes of the wildtype and mutant in case of undisturbed radial growth such that

$$\begin{aligned} \tilde{N}_{\text{wt}} &= \frac{4}{3}\pi x_{\text{wt}}^3(\tau) \\ \Delta_1(\tau) &= \frac{4}{3}\pi x_{\text{m}}^3(\tau). \end{aligned} \quad [3]$$

Further, $\Delta_2(\tau)$ is the intersecting volume of two balls with radii x_{wt} and x_{m} at distance y. Following ref. (1), we have

$$\Delta_2(\tau) = \frac{\pi}{12y}(x_{\text{wt}} + x_{\text{m}} + y)^2(y^2 + 2yx_{\text{m}} - 3x_{\text{m}}^2 + 2yx_{\text{wt}} + 6x_{\text{wt}}x_{\text{m}} - 3x_{\text{wt}}^2). \quad [4]$$

We express x_{wt} and x_{m} in terms of the wildtype radius at the time the first mutant occurred x and the time τ from the emergence of the first mutant,

$$\begin{aligned} x_{\text{wt}}(\tau) &= c_{\text{wt}}t = c_{\text{wt}}(\tau + x/c_{\text{wt}}) \\ x_{\text{m}}(\tau) &= c_{\text{m}}\tau. \end{aligned} \quad [5]$$

Now, we are ready to integrate over $N_{\text{wt}}(\tau)$. We obtain

$$\int_0^\infty N_{\text{wt}}(\tau)\,d\tau = \frac{\pi\left(15x^4\left(3c_{\text{m}}^2 - 3c_{\text{m}}c_{\text{wt}} + c_{\text{wt}}^2\right) - y^4\left(23c_{\text{m}}^2 + 31c_{\text{m}}c_{\text{wt}} + 23c_{\text{wt}}^2\right) - 210c_{\text{m}}^2x^2y^2\right)}{45(c_{\text{m}} - c_{\text{wt}})^3}. \quad [6]$$

Inserting this into eqn. 1 gives us the conditional sweep probability. The exact solution reduces to the solution in the main text with $y = 0$, for which

$$\int_0^\infty N_{\text{wt}}(\tau)\,d\tau = \frac{1}{\mu}\left(\frac{x}{\alpha}\right)^4 \quad \text{with} \quad \alpha = \sqrt[4]{\frac{3(c_{\text{m}} - c_{\text{wt}})^3}{\pi\mu(c_{\text{wt}}^2 - 3c_{\text{wt}}c_{\text{m}} + 3c_{\text{m}}^2)}}. \quad [7]$$

4. The sweep probability is independent of the mutation rate

We show that the unconditional selective sweep probability $\Pr(\text{sweep})$ (eqn. 11 in the main text) is independent of the mutation rate. We start by defining homogeneous functions and stating a lemma for homogeneous functions. We then show how this lemma proves independence of the unconditional sweep probability and eventually prove the lemma. Here, we focus on the three-dimensional case whereas the independence for the two- and one-dimensional case is shown later.

A. Homogeneous functions.

Definition 1. *A function* $f : \mathbb{R}^n \to \mathbb{R}$ *is called homogeneous of degree* k *if*

$$f(sx_1, sx_2, ..., sx_n) = s^k f(x_1, x_2, ..., x_n) \qquad [8]$$

for every $x_1, x_2, ..., x_n$ *and* $s \neq 0$.

Noteworthy, polynomials in which all terms have the same polynomial degree k are homogeneous functions with degree k. For example, $5x^2y^2 + \pi x^4$ is homogeneous in x and y with degree 4.

Lemma 1. *Let* $\nu > 0$. *Let* $H(x, y)$, $P(x, y)$ *and* $Q(x, y)$ *be homogeneous functions in* x *and* y *with degrees* $h, p, q > 0$ *respectively and* $Q(x, y) \neq 0$ *for all* $x > 0$ *and* $y > 0$. *Then, the integral*

$$I = \int_0^\infty \int_0^x e^{-\nu H(x,y)} \frac{P(x, y)}{Q(x, y)} \, dy \, dx \qquad [9]$$

is proportional to $\nu^{\frac{q-p-2}{h}}$.

B. The sweep probability is independent of the mutation rate. To apply the lemma on the unconditional sweep probability, we set $\nu = \mu$ and

$$\begin{aligned} H(x, y) &= \int_0^\infty N_{\text{wt}}(\tau) \, d\tau + \frac{3c_{\text{wt}}}{\pi} x^4 \\ P(x, y) &= 3y^2 \times 4x^3 \\ Q(x, y) &= \frac{3c_{\text{wt}}}{\pi} x^3. \end{aligned} \qquad [10]$$

It is straightforward that $P(x, y)$ and $Q(x, y)$ are homogeneous in x and y with degrees $p = 5$ and $q = 3$. Looking at eqn. 6, it is also apparent that $H(x, y)$ is homogeneous with degree 4. Using those expressions and applying lemma 1, we have

$$\Pr(\text{sweep}) = \mu \times I \propto \mu \times \mu^{\frac{3-5-2}{4}} = \mu^0, \qquad [11]$$

which proves the independence of the mutation rate.

We found that the approximate solution using $f_Y(y|X = x) = \delta(y)$ is also independent of the mutation rate indicating a more general result. Looking at the derivation above, this becomes clear. Consider fixed equations for $\Pr(\text{sweep}|X = x, Y = y)$ and $f_X(x)$. For any density function $f_Y(y|X = x)$ that is homogeneous in x and y with degree -1, we can define functions $P(x, y)$ and $Q(x, y)$ such that their difference in degrees is $p - q = -2$. After the application of lemma 1, it follows independence.

C. Proof of Lemma 1.

Proof of lemma 1. We show the relation by making the substitutions

$$\hat{x} = \nu^{1/h} x \quad \text{and} \quad \hat{y} = \nu^{1/h} y$$

such that

$$\begin{aligned} H(\hat{x}, \hat{y}) &= \nu H(x, y) \\ P(\hat{x}, \hat{y}) &= \nu^{p/h} P(x, y) \\ Q(\hat{x}, \hat{y}) &= \nu^{q/h} Q(x, y) \end{aligned}$$

and

$$dx = \nu^{-1/h} d\hat{x} \quad \text{and} \quad dy = \nu^{-1/h} d\hat{y}.$$

Inserting the substitutions in eqn. 9, we obtain

$$\begin{aligned} I &= \int_0^\infty \int_0^x e^{-\nu H(x,y)} \frac{P(x, y)}{Q(x, y)} \, dy \, dx \\ &= \int_0^\infty \int_0^{\hat{x}} e^{-H(\hat{x},\hat{y})} \frac{\nu^{-p/h} P(\hat{x}, \hat{y})}{\nu^{-q/h} Q(\hat{x}, \hat{y})} \nu^{-1/h} \nu^{-1/h} \, d\hat{y} \, d\hat{x} \\ &= \nu^{-2/h} \nu^{(q-p)/h} \int_0^\infty \int_0^{\hat{x}} e^{-H(\hat{x},\hat{y})} \frac{P(\hat{x}, \hat{y})}{Q(\hat{x}, \hat{y})} \, d\hat{y} \, d\hat{x} \end{aligned}$$

The remaining integral is no longer dependent on parameter ν and shows the desired scaling with $\nu^{\frac{q-p-2}{h}}$. □

5. Sweep probability in one dimension

We consider a population that is expanding in one dimension in two directions with constant speed. If x_{wt} describes the length from the origin of the population to the leading edge, then the population size is $N_{\mathrm{wt}} = 2x_{\mathrm{wt}}(t) = 2c_{\mathrm{wt}}t$. We follow the derivations described in the main text.

A. Arrival time and location of the first mutant. We start by computing the probability density for the time at which the first mutant arises. Using claim 1, we compute the probability that no mutant occurs until time t,

$$P_0 = e^{-\mu\int_0^t 2c_{\mathrm{wt}}t'\,dt'} = e^{-\mu c_{\mathrm{wt}}t^2} = e^{-\left(\frac{t}{\kappa_{1\mathrm{D}}}\right)^2} \quad \text{with} \quad \kappa_{1\mathrm{D}} = \sqrt{\frac{1}{\mu c_{\mathrm{wt}}}}. \qquad [12]$$

The probability density function for the timing of the first surviving mutant is then

$$f_T(t) = \frac{d(1-P_0)}{dt} = \frac{2t}{\kappa_{1\mathrm{D}}^2}e^{-\left(\frac{t}{\kappa_{1\mathrm{D}}}\right)^2}. \qquad [13]$$

With a change of variables $t = \frac{x}{c_{\mathrm{wt}}}$, we obtain the probability density for the population radius X at the time the first surviving mutant arises, which is

$$f_X(x) = \frac{2x}{\theta_{1\mathrm{D}}^2}e^{-\left(\frac{x}{\theta_{1\mathrm{D}}}\right)^2} \quad \text{with} \quad \theta_{1\mathrm{D}} = \sqrt{\frac{c_{\mathrm{wt}}}{\mu}}. \qquad [14]$$

Since all distances y from the wildtype origin have the same amount of proliferating cells, the probability density for the location of the first surviving mutant Y conditional on $X = x$ is given by a homogeneous probability density. After consideration of boundary conditions and normalization, we have

$$f_Y(y|X=x) = \frac{1}{x}1\{y \le x\}, \qquad [15]$$

We compute the unconditional probability density of y,

$$f_Y(y) = \int_0^\infty f_X(x)f_Y(y|X=x)\,dx = \frac{1}{\theta_{1\mathrm{D}}}\Gamma\left(\frac{1}{2}, \frac{y^2}{\theta_{1\mathrm{D}}^2}\right). \qquad [16]$$

B. Exact sweep probability. In one dimension, we obtain a closed-form expression for the exact sweep probability. We start with the sweep probability conditioned on $X = x$ and $Y = y$. The remaining wildtype population is

$$N_{\mathrm{wt}} = (2x_{\mathrm{wt}} - 2x_{\mathrm{m}})\,\mathbf{1}[0,\tau_1] + (x_{\mathrm{wt}} - x_{\mathrm{m}} + y)\,\mathbf{1}[\tau_1,\tau_2] \qquad [17]$$

where $\tau_1 = \frac{x-y}{c_{\mathrm{m}}-c_{\mathrm{wt}}}$ is the time at which the mutant population reaches the first leading edge of the wildtype and $\tau_2 = \frac{x+y}{c_{\mathrm{m}}-c_{\mathrm{wt}}}$ is the time at which the mutant reaches the second leading edge of the wildtype such that the sweep is completed. Next, we substitute $x_{\mathrm{wt}} = x + c_{\mathrm{wt}}\tau$ and $x_{\mathrm{m}} = c_{\mathrm{m}}\tau$ and then apply claim 1 to compute the probability that no further mutant arises before the sweep is completed, which leads to

$$\Pr(\text{sweep}|X=x,Y=y) = e^{-\mu\int_0^\infty N_{\mathrm{wt}}(\tau)\,d\tau} = e^{-\frac{x^2+y^2}{\alpha_{1\mathrm{D}}^2}} \quad \text{with} \quad \alpha_{1\mathrm{D}} = \sqrt{\frac{c_{\mathrm{m}}-c_{\mathrm{wt}}}{\mu}}. \qquad [18]$$

Then, we marginalize out Y, which gives us

$$\Pr(\text{sweep}|X=x) = \int_0^\infty \Pr(\text{sweep}|X=x,Y=y)f_Y(y|X=x)\,dy = \frac{\sqrt{\pi}\alpha_{1\mathrm{D}}}{2x}e^{-\left(\frac{x}{\alpha_{1\mathrm{D}}}\right)^2}\operatorname{erf}\left(\frac{x}{\alpha_{1\mathrm{D}}}\right), \qquad [19]$$

where $\operatorname{erf}(x) = \frac{2}{\sqrt{\pi}}\int_0^x e^{-t^2}\,dt$ is the Gaussian error function. The exact formula for the unconditional sweep probability is obtained by marginalizing out X. The result is

$$\Pr(\text{sweep}) = \int_0^\infty \Pr(\text{sweep}|X=x)f_X(x)\,dx = \frac{\beta'\cot^{-1}\left(\sqrt{1+\beta'}\right)}{\sqrt{1+\beta'}}, \qquad [20]$$

which is independent on the mutation rate. Here, $\beta' = \frac{c_{\mathrm{m}}-c_{\mathrm{wt}}}{c_{\mathrm{wt}}}$ is the speed difference relative to the wildtype speed.

C. Approximate sweep probability. We use the approximation explained in the main text and set $y = 0$, which translates into probability density $f_Y(y|X = x) = \delta(y)$. Under this assumption, the conditional sweep probability is

$$\Pr(\text{sweep}|X = x) = \Pr(\text{sweep}|X = x, Y = 0) = e^{-\left(\frac{x}{\alpha_{1\text{D}}}\right)^2}. \quad [21]$$

and the unconditional sweep probability becomes

$$\Pr(\text{sweep}) = \int_0^\infty \Pr(\text{sweep}|X = x) f_X(x)\, dx = \frac{c_\text{m} - c_\text{wt}}{c_\text{m}}. \quad [22]$$

Using Bayes theorem, we compute the probability distribution of X given a sweep has occurred,

$$f_X(X = x|\text{sweep}) = \frac{2x}{\theta_{1\text{D}}^2 \beta} e^{-\frac{x^2}{\theta_{1\text{D}}^2 \beta}}, \quad [23]$$

where $\beta = \frac{c_\text{m} - c_\text{wt}}{c_\text{m}}$. The distribution of X is still described by a Weibull distribution with shape parameter 2. The difference is only a scaling factor of the characteristic length $\theta_{1\text{D}} \to \theta_{1\text{D}}\sqrt{\beta}$.

Because $y = 0$ minimizes N_wt, eqn. 22 corresponds to an upper bound on the sweep probability. We can likewise set $y = x$ to obtain the lower bound

$$\Pr(\text{sweep}) > \frac{c_\text{m} - c_\text{wt}}{c_\text{m} + c_\text{wt}}. \quad [24]$$

By setting $y^2 = \mathbb{E}[Y^2] = x^2/3$, we further obtain

$$\Pr(\text{sweep}) \approx \frac{c_\text{m} - c_\text{wt}}{c_\text{m} + c_\text{wt}/3}, \quad [25]$$

which is within 5% of the exact result for all $c_m > c_\text{wt}$. A prior approximation for the arctangent function (2) implies that an even better approximation is

$$\Pr(\text{sweep}) \approx \frac{c_\text{m} - c_\text{wt}}{c_\text{m} + 0.28125 c_\text{wt}}, \quad [26]$$

which is always within 1% of the exact solution. The upper bound is never more than 28.125% greater than this latter approximation and their difference decreases monotonically as c_m/c_wt increases.

6. Sweep probability in two dimensions

We consider a population that is expanding radially in two dimensions at constant speed. If x_wt is the radius of the wildtype population, then the population size is $N = \pi x_\text{wt}^2 = \pi(c_\text{wt} t)^2$. We follow the derivations in the main text.

A. Arrival time and location of the first mutant. We start and compute the probability density for the time at which the first mutant arises. Using claim 1, we compute the probability that no mutant occurs until time t,

$$P_0 = e^{-\mu \int_0^t \pi c_\text{wt}^2 t'^2\, dt'} = e^{-\mu \frac{\pi}{3} c_\text{wt}^2 t^3} = e^{-\left(\frac{t}{\kappa_{2\text{D}}}\right)^3} \quad \text{with} \quad \kappa_{2\text{D}} = \sqrt[3]{\frac{3}{\mu \pi c_\text{wt}^2}}. \quad [27]$$

Taking the derivative gives us the probability density for the arrival time of the first surviving mutant,

$$f_T(t) = \frac{d(1 - P_0)}{dt} = \frac{3t^2}{\kappa_{2\text{D}}^3} e^{-\left(\frac{t}{\kappa_{2\text{D}}}\right)^3}. \quad [28]$$

We perform a change of variables $t = \frac{x}{c_\text{wt}}$ to obtain the probability density for the radius of the wildtype population X at the time the first mutant arises that is

$$f_X(x) = \frac{3x^2}{\theta_{2\text{D}}^3} e^{-\left(\frac{x}{\theta_{2\text{D}}}\right)^3}, \quad \text{with} \quad \theta_{2\text{D}} = \sqrt[3]{\frac{3c_\text{wt}}{\pi\mu}}. \quad [29]$$

To compute the probability density for the distance Y between the first mutant and the centre of the wildtype population, we notice that there at distance y, there are $2\pi y\, dy$ dividing cells at distance y. Given that the first mutant occurred when the wildtype population had radius $X = x$, we have $f_Y(y|X = x) \propto y$. After taking care of the boundary condition and normalization, we obtain

$$f_Y(y|X = x) = \frac{2y}{x^2} 1\{y \leq x\}, \quad [30]$$

The unconditional probability density of y is

$$f_Y(y) = \int_0^\infty f_Y(y|X = x) f_X(x)\, dx = \frac{2y}{\theta_{2D}^2} \Gamma\left(\frac{1}{3}, \frac{y^3}{\theta_{2D}^3}\right). \quad [31]$$

B. Exact sweep probability. We start by finding the expression for the remaining wildtype population N_{wt} given that $X = x$ and $Y = y$. As in the three- and one-dimensional cases, we have the remaining wildtype population given by two formulas such that

$$N_{\mathrm{wt}}(\tau) = (\tilde{N}_{\mathrm{wt}}(\tau) - \Delta_1(\tau))\mathbf{1}\{[0,\tau_1]\} + (\tilde{N}_{\mathrm{wt}}(\tau) - \Delta_2(\tau))\mathbf{1}\{[\tau_1,\tau_2]\} \qquad [32]$$

with $\tau_1 = \frac{x-y}{c_{\mathrm{m}}-c_{\mathrm{wt}}}$ and $\tau_2 = \frac{x+y}{c_{\mathrm{m}}-c_{\mathrm{wt}}}$. The population sizes of undisturbed growth are given by the area of discs with radii x_{wt} and x_{m}

$$\begin{aligned}\tilde{N}_{\mathrm{wt}}(\tau) &= \pi x_{\mathrm{wt}}^2 \\ \Delta_1(\tau) &= \pi x_{\mathrm{m}}^2\end{aligned} \qquad [33]$$

and the area of wildtype replaced by the mutant after $\tau > \tau_1$ is given by the intersecting area between two discs with radii x_{wt}, x_{m} at distance y taken from ref. (3) which becomes

$$\begin{aligned}\Delta_2(\tau) =& x_{\mathrm{wt}}^2 \cos^{-1}\left(\frac{y^2 + x_{\mathrm{wt}}^2 - x_{\mathrm{m}}^2}{2yx_{\mathrm{wt}}}\right) + x_{\mathrm{m}}^2 \cos^{-1}\left(\frac{y^2 + x_{\mathrm{m}}^2 - x_{\mathrm{wt}}^2}{2yx_{\mathrm{m}}}\right) \\ &- \frac{1}{2}\big((-y + x_{\mathrm{wt}} + x_{\mathrm{m}})(y + x_{\mathrm{wt}} - x_{\mathrm{m}})(y - x_{\mathrm{wt}} + x_{\mathrm{m}})(y + x_{\mathrm{wt}} + x_{\mathrm{m}})\big)^{1/2}.\end{aligned} \qquad [34]$$

Again, we replace $x_{\mathrm{wt}} = x + c_{\mathrm{wt}}\tau$ and $x_{\mathrm{m}} = c_{\mathrm{m}}\tau$.

Applying claim 1 on the remaining wildtype population, we obtain the conditional sweep probability

$$\Pr(\text{sweep}|X = x, Y = y) = e^{-\mu \int_0^\infty N_{\mathrm{wt}}(\tau)\, d\tau}. \qquad [35]$$

The unconditional sweep probability is then given by the integral

$$\begin{aligned}\Pr(\text{sweep}) &= \int_0^\infty \int_0^\infty \Pr(\text{sweep}|X = x, Y = y) f_Y(y|X = x) f_X(x)\, dy\, dx, \\ &= \int_0^\infty \int_0^x e^{-\mu \int_0^\infty N_{\mathrm{wt}}(\tau) d\tau} \frac{2y}{x^2} \frac{3x^2 e^{-x^2/\theta_{2\mathrm{D}}^3}}{\theta_{2\mathrm{D}}^3}\, dy\, dx,\end{aligned} \qquad [36]$$

which we solved numerically.

C. Independence of the mutation rate. We apply lemma 1 on the integral form of Pr(sweep) by defining $\nu = \mu$ and

$$\begin{aligned}H(x,y) &= \int_0^\infty N_{\mathrm{wt}}(\tau) d\tau + x^3 \frac{3c_{\mathrm{wt}}}{\pi}, \\ P(x,y) &= 2y \times 3x^2, \\ Q(x,y) &= x^2 \frac{\pi}{3c_{\mathrm{wt}}},\end{aligned} \qquad [37]$$

which are homogeneous and have degrees $h = 3$, $p = 3$ and $q = 2$. It follows that

$$\begin{aligned}\Pr(\text{sweep}) &= \mu \times \int_0^\infty \int_0^x e^{-\mu H(x,y)} \frac{P(x,y)}{Q(x,y)}\, dy\, dx \\ &\propto \mu \times \mu^{\frac{2-3-2}{3}} = \mu^0,\end{aligned} \qquad [38]$$

which proves independence of he mutation rate.

D. Approximate sweep probability. We use the approximation explained in the main text and set $y = 0$, which translates into a probability density $f_Y(y|X = x) = \delta(y)$. Under this assumption, the conditional sweep probability is given by

$$\Pr(\text{sweep}|X = x) = \Pr(\text{sweep}|X = x, Y = 0) = e^{-\left(\frac{x}{\alpha_{2\mathrm{D}}}\right)^3}, \quad \text{with} \quad \alpha_{2\mathrm{D}} = \sqrt[3]{\frac{3(c_{\mathrm{m}} - c_{\mathrm{wt}})^2}{\pi\mu(2c_{\mathrm{m}} - c_{\mathrm{wt}})}} \qquad [39]$$

and the unconditional sweep probability becomes

$$\Pr(\text{sweep}) = \left(\frac{c_{\mathrm{m}} - c_{\mathrm{wt}}}{c_{\mathrm{m}}}\right)^2 \qquad [40]$$

Using Bayes theorem, we compute the probability distribution of X given a sweep has occurred,

$$f_X(X = x|\text{sweep}) = \frac{3x^2}{\theta_{2\mathrm{D}}^3 \beta^2} e^{-\frac{x^3}{\theta_{2\mathrm{D}}^3 \beta^2}} \qquad [41]$$

where $\beta = \frac{c_{\mathrm{m}}-c_{\mathrm{wt}}}{c_{\mathrm{m}}}$. The distribution of X is still described by a Weibull distribution with shape parameter 3. The difference is only a scaling factor of the characteristic length $\theta_{2\mathrm{D}} \to \theta_{2\mathrm{D}}\beta^{\frac{2}{3}}$.

7. Sweep probabilities in other growth models

We focused on constant radial expansion speeds in spatial populations for reasons explained in the main text. However, to obtain more generality, we also computed sweep probabilities in other growth models.

A. Constant population with radially expanding mutant. We consider a constant population of spherical form with radius x_0 leading to total population size $N_0 = \frac{4}{3}\pi x_0^3$. The probability distribution of the wildtype radius X at the time the first mutant arises is fixed at x_0 and is formally written as delta function,

$$f_X(x) = \delta(x - x_0). \quad [42]$$

The probability density for the distance Y between the first mutant and the centre of the wildtype population conditioned on $X = x$ is the same as for expanding populations. However, we can replace the random variable $X = x$ by the fixed radius x_0 such that

$$f(y) = f(y|X = x_0) = \frac{3y^2}{x_0^3}\mathbf{1}\{y < x_0\}. \quad [43]$$

Next, we calculate the sweep probability conditioned on X and Y. We denote the remaining wildtype population once a mutant arose by $N_{\mathrm{wt}}(\tau)$ and analogously to the previous calculation introduce the time measure τ that starts with the emergence of the first mutant. We have

$$N_{\mathrm{wt}} = (\tilde{N}_{\mathrm{wt}}(\tau) - \Delta_1(\tau))\mathbf{1}\{[0, \tau_1]\} + (\tilde{N}_{\mathrm{wt}}(\tau) - \Delta_2(\tau))\mathbf{1}\{[\tau_1, \tau_2]\} \quad [44]$$

where the terms are identical to those described in section 3 except for constant wildtype radius x_0,

$$\begin{aligned} \tilde{N}_{\mathrm{wt}} &= \frac{4}{3}\pi x_0^3 \\ \Delta_1(\tau) &= \frac{4}{3}\pi x_{\mathrm{m}}^3 \\ \Delta_2(\tau) &= \frac{\pi}{12y}(x_0 + x_{\mathrm{m}} + y)^2(y^2 + 2yx_{\mathrm{m}} - 3x_{\mathrm{m}}^2 + 2yx_0 + 6x_0x_{\mathrm{m}} - 3x_0^2), \end{aligned} \quad [45]$$

and $x_{\mathrm{m}} = x_{\mathrm{m}}(\tau) = c_{\mathrm{m}}\tau$. Using claim 1, the conditional sweep probability is computed by

$$\Pr(\text{sweep}|Y = y) = e^{-\mu\int_0^\infty N_{\mathrm{wt}}(\tau)\,d\tau} \quad \text{with} \quad \int_0^\infty N_{\mathrm{wt}}(\tau)\,d\tau = \frac{\pi\left(45x_0^4 - 210x_0^2y^2 - 23y^4\right)}{45c_{\mathrm{m}}}. \quad [46]$$

Integrating the conditional sweep probability together with $f_Y(y)$ does not yield a closed-form expression.

For analytical insight, let us assume again that the mutant originates in the centre of the wildtype population, $f_Y(y) = \delta(y)$. We then obtain

$$\Pr(\text{sweep}) = \Pr(\text{sweep}|Y = 0) = e^{-\frac{\mu\pi x_0^4}{c_m}}. \quad [47]$$

We compared this sweep probability for different x_0 to sweep probabilities in expanding populations (Figure 5).

Performing the same analysis in one and two dimensions, we obtain

$$\begin{aligned} \Pr(\text{sweep}) &= e^{-\frac{\mu x_0^2}{2c_{\mathrm{m}}}} \quad \text{in 1D} \\ \Pr(\text{sweep}) &= e^{-\frac{2\mu\pi x_0^3}{3c_{\mathrm{m}}}} \quad \text{in 2D.} \end{aligned} \quad [48]$$

Comparison with Ralph & Coop: Motivated by parallel adaptation on the species level, Ralph & Coop investigated selective sweeps in constant populations using a similar approach (4). However, whereas we assume spherical wildtype populations, Ralph & Coop consider a general shapes and use scaling arguments. To derive a concrete expression, they ignore boundary effects and obtain "the expected number of other mutations to arise in an area of diameter a in the time it takes the wave to cover that area". Simplifying eqn. (4) in ref. (4) and keeping their notation, we have

$$\frac{2a^3\lambda}{v} \quad \text{in 1D} \quad \text{and} \quad \frac{\pi a^3\lambda}{v} \quad \text{in 2D.} \quad [49]$$

The number of mutations arising in an area over time is a Poisson process (claim 1). Thus, we can interpret this number as mean of a Poisson distribution. A selective sweep has occurred if no other mutation arose and thus the sweep probability reads

$$\begin{aligned} \Pr(\text{sweep}) &= P_0 = e^{-\frac{2a^2\lambda}{v}} \quad \text{in 1D,} \\ \Pr(\text{sweep}) &= P_0 = e^{-\frac{\pi a^3\lambda}{v}} \quad \text{in 2D.} \end{aligned} \quad [50]$$

We identify v as the speed of the mutant c_{m}, λ as the local mutation rate conditioned on survival $\mu = \tilde{\mu}\rho$ and a as the maximal travelled distance by the first mutant, which is x_0 in the case we set $y = 0$. Finally, we find the solution of Ralph & Coop (eqn. 50) to be identical to our solution (eqn. 48) up to multiplication by a constant that can be explained by differing boundary conditions. The conclusion in either case is that full selective sweeps are highly unlikely for sufficiently large population radius x_0.

Comparison with Martens et al.: Martens and colleagues investigated the likelihood of clonal interference and its impact on the speed of evolution in spatially structured constant-size populations (5), and then studied the implications for understanding cancer initiation (6). Two modes of evolution are considered: (i) acquisition via subsequent selective sweeps and (ii) acquisition with parallel arising mutations which interfere with each other. To distinguish between these two modes, the authors compare the timescale for a surviving mutation to occur, t_{mut}, and the timescale for a mutation to sweep through the entire constant wildtype population, t_{fix}. Equating these two timescales leads to a critical length of the wildtype population:

$$\begin{aligned} L_c &= \left(\frac{c_0}{2s_0\mu}\right)^{\frac{1}{2}} \quad \text{in 1D} \\ L_c &= \left(\frac{c_0}{2s_0\mu}\right)^{\frac{1}{3}} \quad \text{in 2D.} \end{aligned} \tag{51}$$

The authors argue that clonal interference is very likely in populations of size $L \gg L_c$, whereas we should expect selective sweeps when $L \ll L_c$. To compare this result with our model, we put L_c into eqn. 48. Therefore, we match parameters by $c_0 \leftrightarrow c_{\text{m}}$, $2s\mu \leftrightarrow \rho\tilde{\mu} = \mu$ and $x_0 \leftrightarrow L$ leading to

$$\begin{aligned} \Pr(\text{sweep}) &= e^{-\frac{x_0^2}{2L_c^2}} \quad \text{in 1D} \\ \Pr(\text{sweep}) &= e^{-\frac{\pi x_0^3}{3L_c^3}} \quad \text{in 2D.} \end{aligned} \tag{52}$$

Indeed, we have $\Pr(\text{sweep}) \to 0$ for $x_0 \gg L_c$ and $\Pr(\text{sweep}) \to 1$ for $x_0 \ll L_c$. We conclude that the result of Martens et al. are in agreement with our result for constant population sizes.

B. Exponential growth. We consider an exponentially growing population, $N(t) = e^{rt}$. Applying claim 1, the probability that no mutants occur until time t is given by

$$P_0 = e^{-\mu\int_0^t e^{rt'}\,dt'} = e^{-\frac{\mu}{r}(e^{rt}-1)}. \tag{53}$$

Dropping the -1, this equation coincides with the solution for the stochastic birth-death process obtained by Durrett (eqn. [25] in Ref. (7)) up to the constant V_0 that is caused by fluctuations in early growth history and is neglected within our deterministic growth assumption. We proceed to calculate the probability density of T by

$$f(t) = \frac{d(1-P_0)}{dt} = \mu e^{rt} e^{-\frac{\mu}{r}e^{rt}}, \tag{54}$$

Instead of asking for the radius, it is more sensible to ask for the population size of the wildtype at the arrival of the first mutant. We write $N_x = e^{rt}$, such that $t = \frac{1}{r}\ln(N_x)$. After substitution, we have

$$f_{N_x}(N_x) = \frac{\mu}{r} e^{-\frac{\mu}{r}N_x}, \tag{55}$$

which is an exponential distribution. The probability for the radius X can then be calculated by assuming spherical growth starting from one cell, $N_x = \frac{4}{3}\pi x + 1$. For mathematical convenience, we neglect the $+1$ term. The probability distribution of X reads

$$f_X(x) = \frac{3x^2}{\theta_{\text{exp}}^3} e^{-\frac{x^3}{\theta_{\text{exp}}^3}} \quad \text{with} \quad \theta_{\text{exp}} = \sqrt[3]{\frac{3r}{4\pi\mu}}. \tag{56}$$

Exponential growth does not assume a particular spatial structure and the dispersal mode of the mutant is unclear. Nevertheless, no such assumptions are required to compute the location at which the first mutant arises. The conditional probability distribution of Y remains the same as in our main model, that is

$$f_Y(y|X=x) = \frac{3y^2}{x^3} 1\{y \le x\}. \tag{57}$$

To obtain the unconditional probability for y, we can marginalize out X giving us

$$\begin{aligned} f_Y(y) &= \int_0^\infty f_Y(y|X=x) f_X(x)\,dx \\ &= \frac{9y^2}{\theta_{\text{exp}}^3}\int_y^\infty \frac{e^{-\frac{x^3}{\theta_{\text{exp}}^3}}}{x}\,dx \\ &= \frac{3y^2}{\theta_{\text{exp}}^3}\text{Ei}\left(\frac{x^3}{\theta_{\text{exp}}^3}\right), \end{aligned} \tag{58}$$

where $\text{Ei}(z) = \int_0^\infty \frac{1}{t}e^{-t}\,dt$ is te exponential integral function.

In the exponential growth model, there is no competition such that the wildtype population will never go extinct and the mutant will never become fixed. We can however compute whether the mutant reaches a certain frequency χ before another mutant arises. We assume deterministic growth of the wildtype and the mutant and random time for the emergence of the mutant. Denoting the wildtype population size by N_{wt} and mutant population size by N_{m}, we have

$$\chi = \frac{N_{\mathrm{m}}}{N_{\mathrm{m}} + N_{\mathrm{wt}}}. \qquad [59]$$

Now, define t_1 to be the time at which the first mutant occurs and let t_2 be the time at which the mutant has grown to frequency χ. Then

$$\chi = \frac{e^{r_{\mathrm{m}} t_2}}{e^{r_{\mathrm{m}} t_2} + e^{r_{\mathrm{wt}}(t_1+t_2)}}. \qquad [60]$$

Here, t_1 is a random variable that follows the probability density described by eqn. 54, and t_2 can be computed by solving the equation for χ giving us

$$t_2 = \frac{r_{\mathrm{wt}} t_1 - \ln\left(\frac{1-\chi}{\chi}\right)}{r_{\mathrm{m}} - r_{\mathrm{wt}}}. \qquad [61]$$

The conditional sweep probability (or more precisely the probability for the first mutant to reach frequency χ without interference of another mutant) is given by

$$\Pr(\text{sweep}|T = t_1) = e^{-\lambda} \quad \text{with} \quad \lambda = \mu \int_0^{t_2} e^{r_{\mathrm{wt}}(t_1+\tau)}\, d\tau = \frac{\mu}{r_{\mathrm{wt}}} e^{r t_1}\left(e^{r_{\mathrm{wt}} t_2} - 1\right), \qquad [62]$$

where $t_2 = t_2(t_1)$ is a function of t_1 described above. Eventually, the unconditional sweep probability is computed by

$$\begin{aligned} \Pr(\text{sweep}) &= \int_0^\infty \Pr(\text{sweep}|T = t_1) \times f_T(t_1)\, dt_1 \\ &= \int_0^\infty e^{-\frac{\mu}{r_{\mathrm{wt}}} e^{r t_1}\left(e^{r_{\mathrm{wt}} t_2(t_1)} - 1\right)} \times \mu e^{r t_1} e^{-\frac{\mu}{r} e^{r t_1}}\, dt_1 \end{aligned} \qquad [63]$$

which we solved numerically and present in Figure 5.

C. Boundary growth with proliferation restricted to the boundary. Antal and colleagues studied aspects of the evolutionary dynamics in boundary-driven growth where cell proliferation is restricted to the boundary only (8). More properties of this system have further been studied extensively (9). Whereas we assume turnover of the entire population, Antal et al. consider turnover only at the boundary and focus on the three-dimensional case. A full selective sweep is impossible in this model since individuals located away from the boundary neither proliferate nor die. Instead, we can consider the probability that a mutant will envelop the wildtype and thus becomes the only proliferating population. Using this interpretation, eqn. (17) in ref. (8) provides the unconditional sweep probability that is

$$\Pr(\text{sweep}) = \frac{9 + \beta^2}{\beta^2} \frac{2}{1 + e^{3\pi/\beta}}, \qquad [64]$$

with $\beta = \sqrt{v^2 - 1}$ and we can identify $v = \frac{c_{\mathrm{m}}}{c_{\mathrm{wt}}}$. The unconditional sweep probability is independent of the mutation rate just like in our model. Furthermore, Antal et al. find similar expressions for the arrival time of the first mutant $f_T(t)$ (eqn. 15 in (8)) and the conditional sweep probability $\Pr(\text{sweep}|X = x)$ (eqn. 16 in ref. (8)) as well as the size of the wildtype population when the first mutant occurs (eqn. 25 in ref. (8) provides the cumulative density function). The unconditional sweep probability in the case of boundary-restricted proliferation is lower compared to the sweep probability allowing proliferation throughout the tumour including the interior (Figure 4).

D. Constant population with logistically growing mutant population. In a well-mixed constant population, it is reasonable to assume logistic growth for the mutant. This case was considered by Gerrish and Lenski (10) who used a similar methodology to ours. If the mutant has selective advantage s over the wildtype, the growth of the mutant starting from a single cell can be written as

$$N_{\mathrm{m}}(t) = \frac{N_0 e^{st}}{N_0 - (1 - e^{st})}. \qquad [65]$$

Here N_0 is the total population size acting as carrying capacity. The remaining wildtype population is $N_{\mathrm{wt}}(t) = N_0 - N_{\mathrm{m}}(t)$. If μ is the mutation rate for beneficial mutations that survive drift, then there are $\mu N_{\mathrm{wt}}(t)\, dt$ mutants generated at time t and the likelihood that at least one interfering mutant arises follows from the application of claim 1. The probability for a successful sweep is

$$\Pr(\text{sweep}) = e^{-\lambda} \quad \text{with} \quad \lambda = \int_0^{t_{\mathrm{end}}} \mu N_{\mathrm{wt}}(t)\, dt, \qquad [66]$$

where t_{end} defines the time at which the sweep has completed have there been no interfering mutants. In logistic growth, the carrying capacity is only approached asymptotically, thus we define t_{end} as the time when all cells except 1 is replaced, that is

$$N_{\text{m}}(t_{\text{end}}) = N_0 - 1 \quad \Rightarrow \quad t_{\text{end}} = \frac{2}{s}\ln(N_0 - 1). \tag{67}$$

Putting this expression into eqn. 66, we obtain $\lambda = \mu\frac{N_0}{s}\ln(N_0 - 1) \approx \mu\frac{N_0}{s}\ln(N_0)$, and the sweep probability becomes

$$\Pr(\text{sweep}) = N_0 e^{-\mu\frac{N_0}{s}}. \tag{68}$$

This formula is a simplified version of eqn. 3 in ref.(10). Gerrish and Lenski assumed that the selective advantage of mutants is exponentially distributed and considered interference only if the interfering mutants had higher fitness than the primary mutant. We have instead assumed that all mutants have the same fitness, and we absorbed the survival of drift into the mutation rate.

E. Sigmoidal growth. Because biological systems cannot grow indefinitely, several models have been developed to describe the dynamics of a population that initially grows exponentially but is bounded by a carrying capacity (11). The most common sigmoidal growth model is logistic growth. If there are at least two subpopulations with different growth parameters, the logistic growth model can be naturally extended to a competitive Lotka-Volterra system. Closed-form expressions are typically not obtained. Furthermore, the competition can be implemented in several ways. A fitness advantage in Lotka-Volterra systems can be obtained by an increased growth rate, an increased carrying capacity, or frequency-dependent competition factors that are the subject of evolutionary game theory (e.g. (12)). A study of selective sweeps in sigmoidal growth models thus comes with further complications that cannot be solved with methods presented in this paper alone.

Nevertheless, until the first mutant arises, there is only one population type (and thus no complicated competition between different types). We can thus compute the time and location of the first surviving mutant. We consider a population that grows logistically according to $N(t) = \frac{K}{1+Ke^{-rt}}$. We apply claim 1, and obtain the probability no mutation occurs until time t,

$$P_0 = e^{-\frac{\mu K}{r}\ln\left(\frac{e^{rt}+K}{K+1}\right)}. \tag{69}$$

The probability density is then obtained by taking the derivative,

$$f_T(t) = \frac{d(1-P_0)}{dt} = \frac{\mu K(K+1)^{\frac{\mu K}{r}}e^{rt}}{(e^{rt}+K)^{\frac{\mu K}{r}+1}}. \tag{70}$$

The probability density for the population size N_x at the time the first mutant arises can be obtained by substituting $N_x = \frac{K}{1+Ke^{-rt}}$ yielding

$$f_{N_x}(N_x) = \frac{\mu\,(K+1)^{\frac{\mu K}{r}}\,(K-N_x)^{\frac{\mu K}{r}-1}}{rK^{\frac{2\mu K}{r}-1}}. \tag{71}$$

Assuming spherical growth, $N_x = \frac{4}{3}\pi x^3$, the probability density for the radius X can be obtained by substituting $N_x = \frac{4}{3}\pi x^3$. We obtain

$$f_X(x) = \frac{\mu\,(K+1)^{\frac{\mu K}{r}}\left(K - \frac{4\pi x^3}{3}\right)^{\frac{\mu K}{r}-1} 4\pi x^2}{rK^{\frac{2\mu K}{r}-1}}. \tag{72}$$

The distance Y of the first mutant from the wildtype's origin conditioned on the radius $X = x$ remains the same as before,

$$f_Y(y|X=x) = \frac{3y^2}{x^3}1\{y \leq x\}. \tag{73}$$

To obtain the unconditional density for Y, we can marginalize out X,

$$f_Y(y) = \int_0^\infty f_Y(y|X=x)f_X(x)\,dx. \tag{74}$$

which gives us

$$\frac{12\pi y^2\mu(K+1)^{\frac{\mu K}{r}}}{rK^{\frac{2\mu K}{r}-1}}\int_y^\infty \frac{\left(K - \frac{4\pi x^3}{3}\right)^{\frac{\mu K}{r}-1}}{x}\,dx. \tag{75}$$

The integral can be evaluated numerically.

F. Sweep probability at 50% . To compare the models in the space of parameters (μ, c_{m}), we can compute the tuples (μ, c_{m}) at which the sweep probability is 50%. The tuples can be expressed as curve $c_{\mathrm{m}}(\mu)$ for which $\Pr(\text{sweep}) = 0.5$ leading to the following expressions for the three-dimensional expansions:

- Growth throughout (eqn. 14 in the main text): $c_{\mathrm{m}}(\mu) \approx 6.3\, c_{\mathrm{wt}}$
- Boundary proliferation (eqn. 64): $c_{\mathrm{m}}(\mu) \approx 7.2\, c_{\mathrm{wt}}$
- constant population (eqn. 47): $c_{\mathrm{m}}(\mu) = \frac{\mu \pi x_0^4}{\ln(2)}$.

We solved the first two expressions numerically.

8. Dependence of the sweep probability on the mutation rate

As explained previously, the mutation rate in an expanding population has two opposing effects on the sweep probability:

1. Higher mutation rate → earlier arrival of the first surviving mutant → smaller wildtype population for the mutant to sweep through → higher sweep probability.
2. Higher mutation rate → earlier arrival of the second, interfering mutant → lower sweep probability.

In models with fixed population size, effect 1 does not apply, so effect 2 causes the sweep probability to decrease with increasing mutation rate. More precisely, we find $\Pr(\text{sweep}) \propto e^{-\text{const.}\ \mu}$ in the spatial and non-spatial cases.

In expanding populations, both effects apply. In the spatial models with constant radial expansion speed, we find that sweep probability is independent of mutation rate, implying that the two opposing effects cancel out. For exponential growth, the sweep probability (or more accurately the probability for the mutant becoming dominant before an interfering mutant arises) increases with mutation rate, implying that effect 1 outweighs effect 2. More precisely, in the exponential case we find $\Pr(\text{sweep}) \propto \mu e^{-\text{const.}\ \mu}$, which is increasing for sufficiently small μ. This makes intuitive sense because arising early is of the greatest advantage when the population growth rate increases rapidly over time.

9. Analysis of selective sweep probability versus mean relative fitness difference at simulation end

Here we consider an alternative way of summarising results of simulations with random mutation fitness effects. Whereas Figure 5C shows selective sweep probability versus the mean effect of surviving mutations, Figure S5 shows the same data in terms of the mean relative fitness difference at the end of simulation. In the latter case we calculate the mean of

$$\frac{a_{\mathrm{x}}}{a_{\mathrm{wt}}} = \frac{r_{\mathrm{x}} - r_{\mathrm{wt}}}{r_{\mathrm{wt}} - r_{\mathrm{re}}}$$

across all individuals when each simulation finishes, where r_{x} is either r_{wt} (if the individual belongs to the wildtype, in which case $a_{\mathrm{x}} = 0$) or r_{m} (if the individual is a mutant). Because the proliferation rates r_{wt} and r_{re} are constant, the mean of $a_{\mathrm{m}}/a_{\mathrm{wt}}$ is simply a linear transformation of the mean proliferation rate across all wildtype and mutant individuals. In Figure S5A, we present the mean value of $a_{\mathrm{x}}/a_{\mathrm{wt}}$ for each batch of simulations that have identical values of the expected mutant fitness effect, s. In Figure S5B, we instead combine results from all simulation batches, spanning multiple s parameter values. We assign simulations to bins according to the mean value of $a_{\mathrm{x}}/a_{\mathrm{wt}}$ and calculate the proportion of simulations that resulted in a selective sweep per bin. The number of simulations per bin varies. Pooling results across different s values might correspond, for example, to pooling tumour data from a diverse patient cohort.

Both plots show that when mutation effects are random and individuals can acquire at most one mutation, the simulation results (red points) are similar to our analytical predictions for the equal-mutation-effects model with the same value of $a_{\mathrm{m}}/a_{\mathrm{wt}}$ (green curves). The main difference between the two plots is that Figure S5B shows a spike in selective sweep frequency associated with very low mean relative fitness of all mutants at the end of the simulation. The spike is not simply due to the pooling of results because it also appears in single-batch results (Figure S5C).

A. Explaining the spike in sweep frequency associated with very low final relative fitness. Figure S5B shows that for simulations with random mutation effects, compared to the equal-mutation-effects model, the probability of a sweep at every final fitness value is higher, and the probability of a sweep at the very lowest fitness values increases from close to zero to a large proportion. Here we explain why this difference is mostly due to a reduction in the number of non-sweeps associated with very low final fitness.

In the macroscopic model, we assume that all mutations confer the same fitness advantage. Hence if two such mutations occur and survive within the wildtype population then the result must be clonal interference, not a sweep. In the agent-based model with random fitness effects, the second and subsequent mutations that arise and survive in the wildtype population may instead confer higher fitness than the first mutation. This makes it possible for the second-arising mutant (or a subsequent mutant) to achieve a sweep by replacing both the wildtype and the first mutant. This outcome is most likely when the first mutant has only a very small fitness advantage over the wildtype.

For simplicity, consider the case when exactly two mutants are competing within the wildtype population and no further mutations occur. We then have three possible scenarios:

- If the second mutant is much less fit than the first then the first mutant is likely to achieve a sweep and the mean mutant fitness at the end of the simulation will be equal to the first mutant's fitness, as in the equal-mutation-effects model.
- If the two mutants are of similar fitness then the outcome will be clonal inference and the mean mutant fitness at the end of the simulation will be similar to the first mutant's fitness, as in the equal-mutation-effects model.
- If the second mutant is much fitter than the first then the second mutant is likely to achieve a sweep by replacing both the wildtype and the first mutant. The mean mutant fitness at the end of the simulation will be equal to the second mutant's fitness. Hence what would, in the equal-mutation-effects model, have been a non-sweep associated with very low final fitness is instead a sweep associated with high fitness.

In summary, the first and second scenarios give the same result as in the equal-mutation-effects model, but the third scenario has a different outcome when the first mutant has very low fitness, leading to fewer non-sweeps associated with very low final fitness.

B. A simple mathematical analysis. To further our understanding of the different outcomes of the equal-mutation-effects and random-mutation-effects models, let us consider the simplest toy model with two initial assumptions:

- No mutant can acquire more than one mutation and no more than two mutants can arise
- With probability $\delta \ll 1$, a mutation confers a very small fitness effect ("weak"); otherwise it confers a large fitness effect ("strong").

We assign outcomes (sweep or non-sweep) to bins (columns) according to the mean mutant fitness at an advanced time, with N_w outcomes in the weak column and N_s outcomes in the strong column. Suppose that our equal-mutation-effects model predicts $\Pr(\text{sweep}) = \epsilon \ll 1$ in the weak column and $\Pr(\text{sweep}) = p$ in the strong column. Then according to our equal-mutation-effects model, which assumes that if two mutants arise then the outcome must be a non-sweep, we predict

- ϵN_w sweeps in the weak column
- $(1-\epsilon)N_w$ non-sweeps in the weak column
- pN_s sweeps in the strong column
- $(1-p)N_s$ non-sweeps in the strong column.

Now assume that if one mutant (either the first or the second) is strong and the other is weak then the strong mutant always achieves a sweep, but if both mutants are weak or both are strong then the result is a non-sweep. Then, accounting for the fitness of the second mutant being random:

- Weak column sweep count remains ϵN_w
- Weak column non-sweep count decreases to $\delta(1-\epsilon)N_w \approx \delta N_w$
- Strong column sweep count increases to $pN_s + (1-\delta)(1-\epsilon)N_w + (1-p)\delta N_s \approx pN_s + N_w$
- Strong column non-sweep count decreases to $(1-p)(1-\delta)N_s \approx (1-p)N_s$.

Now if we assume that the first mutant's fitness is drawn from the same distribution as the second mutant then $N_w = \delta N$ and $N_s = (1-\delta)N$, where $N = N_w + N_s$. Therefore

- $\Pr(\text{sweep})$ in the weak column has increased from ϵ to approximately $\epsilon/(\epsilon+\delta)$; that is, it has increased by factor of approximately $1/(\epsilon+\delta)$, where both ϵ and δ are very small
- $\Pr(\text{sweep})$ in the strong column has changed relatively little, from p to approximately $(pN_s + N_w)/(N_s + N_w) \approx p$.

The outcomes of this model can be expressed in the form of a vector multiplied by a transition matrix:

$$\begin{pmatrix} \delta\epsilon & \delta(1-\epsilon) & (1-\delta)p & (1-\delta)(1-p) \end{pmatrix} \begin{pmatrix} 1 & 0 & 0 & 0 \\ 0 & \delta & 1-\delta & 0 \\ 0 & 0 & 1 & 0 \\ 0 & 0 & \delta & 1-\delta \end{pmatrix}.$$

Here, the vector represents the proportion of outcomes that are sweeps by a weak mutant, non-sweeps by a weak mutant, sweeps by a strong mutant, and non-sweeps by a strong mutant, respectively, assuming that if two mutants arise then the result must be a non-sweep. Multiplying this vector by the transition matrix adjusts the results by accounting for sweeps by the second mutant.

Figure S6 shows results of extending this simple model to the case of five fitness values, using the following transition matrix:

$$\begin{pmatrix}1 & 0 & 0 & 0 & 0 & 0 & 0 & 0 & 0 & 0\\ 0 & q_1 & q_2 & 0 & q_3 & 0 & q_4 & 0 & q_5 & 0\\ 0 & 0 & 1 & 0 & 0 & 0 & 0 & 0 & 0 & 0\\ 0 & 0 & q_1 & q_2 & q_3 & 0 & q_4 & 0 & q_5 & 0\\ 0 & 0 & 0 & 0 & 1 & 0 & 0 & 0 & 0 & 0\\ 0 & 0 & 0 & 0 & q_1+q_2 & q_3 & q_4 & 0 & q_5 & 0\\ 0 & 0 & 0 & 0 & 0 & 0 & 1 & 0 & 0 & 0\\ 0 & 0 & 0 & 0 & 0 & 0 & q_1+q_2+q_3 & q_4 & q_5 & 0\\ 0 & 0 & 0 & 0 & 0 & 0 & 0 & 0 & 1 & 0\\ 0 & 0 & 0 & 0 & 0 & 0 & 0 & 0 & q_1+q_2+q_3+q_4 & q_5\end{pmatrix},$$

where $(q_1, q_2, q_3, q_4, q_5)$ is the distribution of fitness effects. As expected, the figure shows a very large relative increase in sweep frequency associated with the very low fitness class and smaller relative increases associated with higher fitness values. This illustrative model assumes that when two mutants of unequal fitness compete, the outcome will always be a sweep by the fitter mutant. The model will tend to overestimate sweep frequencies because it ignores two alternative outcomes: that the less-fit mutant will slow the fitter mutant's expansion sufficiently to prevent a sweep, or that further mutants arising within the wildtype will prevent a sweep.

10. Sequential mutations

Consider the case in which multiple fitness-modifying mutations can accumulate. Let $c_{\mathrm{m},k}$ denote the propagation speed of mutant populations that have accumulated k mutations, with $c_{\mathrm{m},0} = c_{\mathrm{wt}}$. We will assume $c_{\mathrm{m},k} > c_{\mathrm{m},k-1}$ for $k \geq 1$ as selective sweeps are excluded otherwise. We will also assume that no mutant can acquire more than K mutations.

A. A general upper bound. To obtain an upper bound on the sweep probability, we start by assuming that the first surviving mutant rapidly accumulates the maximum possible number of mutations via soft or hard sweeps. This mutant population, in which the first surviving mutation is clonal, then propagates at speed $c_{\mathrm{m},K}$. Following our previous analysis, the probability that this population sweeps through the wildtype before another competing mutant arises satisfies

$$\Pr(\text{sweep}) < \left(1 - \frac{c_{\mathrm{m},0}}{c_{\mathrm{m},K}}\right)^d. \quad [76]$$

This is only an upper bound for the sweep probability for three reasons. First, we use the approximate rather than the exact sweep probability derived from our single-mutation model. Second, we assume that the first mutant rapidly accumulates the maximum possible number of mutations, which increases the likelihood of a sweep. Third, we neglect that competing mutants may have lower fitness than the first arising mutant and may therefore fail to prevent a sweep.

Similarly, the probability of a population with k mutations achieving a sweep in a population with $k-1$ mutations is bounded by $\left(1 - \frac{c_{\mathrm{m},k-1}}{c_{\mathrm{m},K}}\right)^d$. We thus obtain an upper bound for the probability of having j distinct selective sweeps:

$$\begin{aligned}\Pr(j \text{ sweeps}) = {} & \Pr(\text{1st mutation clonal})\\ & \times \Pr(\text{2nd mutation clonal} \mid \text{1st mutation clonal})\\ & \times \ldots\\ & \times \Pr(j\text{-th mutation clonal} \mid \text{(j-1)th mutation clonal})\\ & < \prod_{k=0}^{j-1}\left(1 - \frac{c_{\mathrm{m},k}}{c_{\mathrm{m},K}}\right)^d.\end{aligned} \quad [77]$$

Because we repeatedly used eqn. 76, the upper bound rests on the same set of assumptions. The bound is closer to the exact probability when j is small or when the maximum propagation speed is not much greater than other mutant propagation speeds.

B. Two mutations with equal effects. Let us now consider the special case in which individuals can acquire at most two mutations with equal multiplicative effects. That is, $r_{m,1} = (1+s)r_{m,0}$ and $r_{m,2} = (1+s)r_{m,1} = (1+s)^2 r_{m,0}$. Combining eqn. 76 together with the scaling from the Fisher speed (akin to eqn. 13 in the main text), we have

$$\Pr(\text{sweep}) \leq \left(1 - \frac{c_{m,0}}{c_{m,2}}\right)^d \approx \left(1 - \sqrt{\frac{r_{m,0} - r_{re}}{r_{m,2} - r_{m,0}}}\right)^d = \left(1 - \sqrt{\frac{r_{m,0} - r_{re}}{r_{m,1} - r_{m,0}}} \times \frac{1}{\sqrt{2+s}}\right)^d. \quad [78]$$

In the second equality we used $r_{m,2} - r_{m,0} = (1+s)^2 r_{m,0} - r_{m,0} = s r_{m,0} \times (2+s)$ and $r_{m,1} - r_{m,0} = s r_{m,0}$ such that $r_{m,2} - r_{m,0} = (r_{m,1} - r_{m,0}) \times (2+s)$.

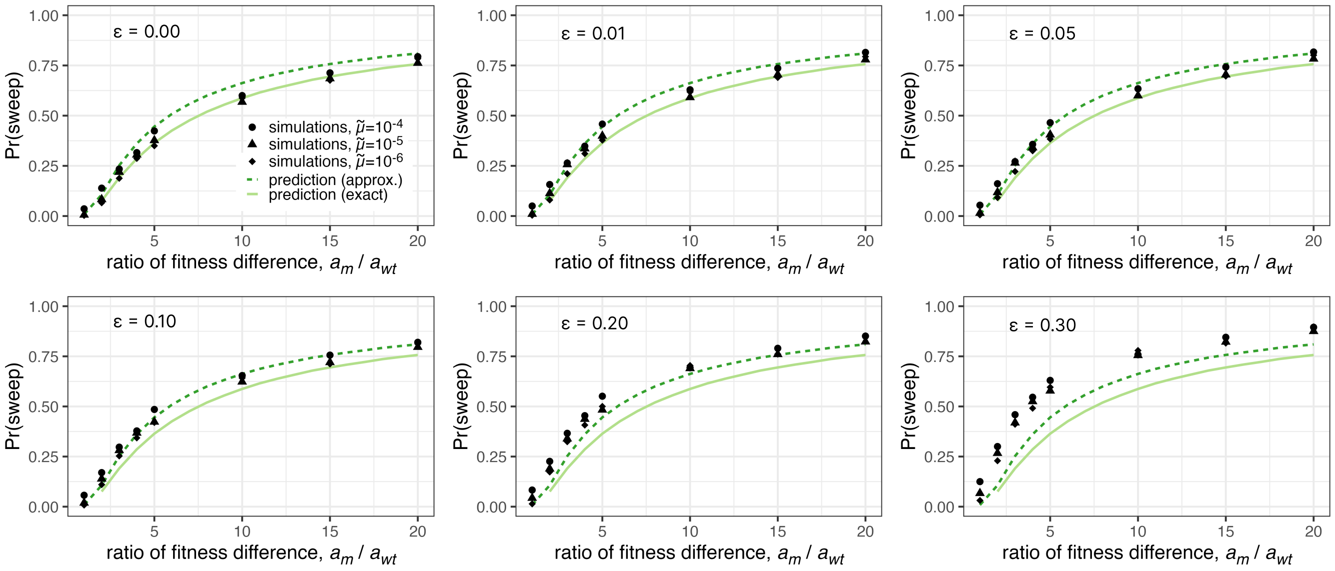

Fig. S1. Simulation results of selective sweep frequencies with different cutoffs in 2D. A selective sweep might be incomplete because of other minority populations. Here, we show simulation results using a weakened definition of a selective sweep. A selective sweep is said to be successful if the mutant's frequency reaches $1-\epsilon$. The case $\epsilon = 0.00$ corresponds to a complete selective sweep as shown and discussed in the main text. Parameters were set to $m = 0.05$, $K = 16$, $\tilde{\mu} = 10^{-4}, 10^{-5}, 10^{-6}$, $r_{\text{re}} = 0.91$, $r_{\text{wt}} = 1.0$. We vary over r_{m} between 1.1 and 3.0 corresponding to different a_{m}. Conversion between simulation and macroscopic model parameters is described in the Materials and Methods.

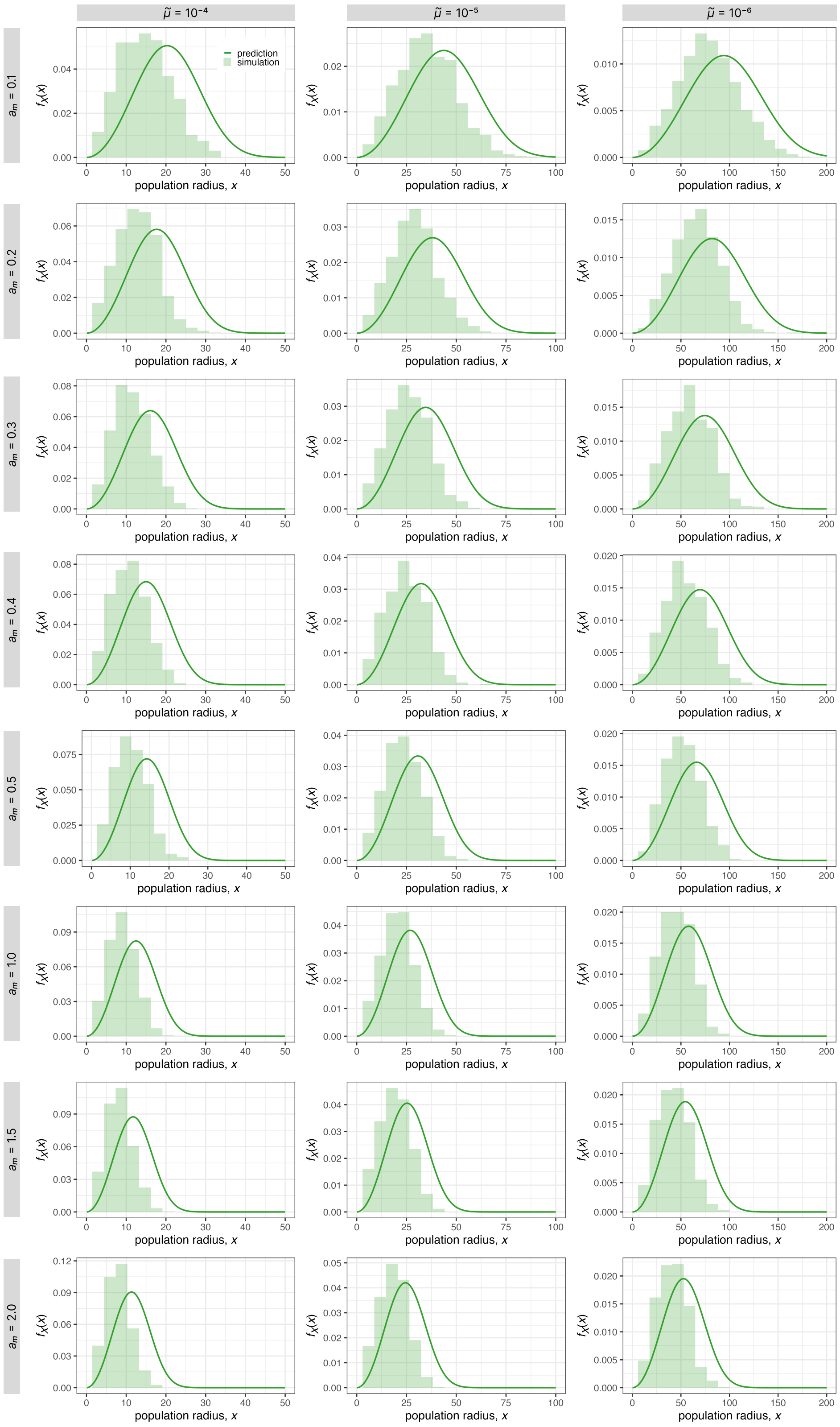

Fig. S2. Comparison of simulation results with our analytic predictions of $f_X(x)$ in 2D for different parameter combinations. We use $m = 0.05$, $K = 16$, $r_{\text{re}} = 0.91$, $r_{\text{wt}} = 1.00$. We vary over the mutation rate μ and r_{m} corresponding to different a_m. Conversion between simulation and macroscopic model parameters is described in the Materials and Methods.

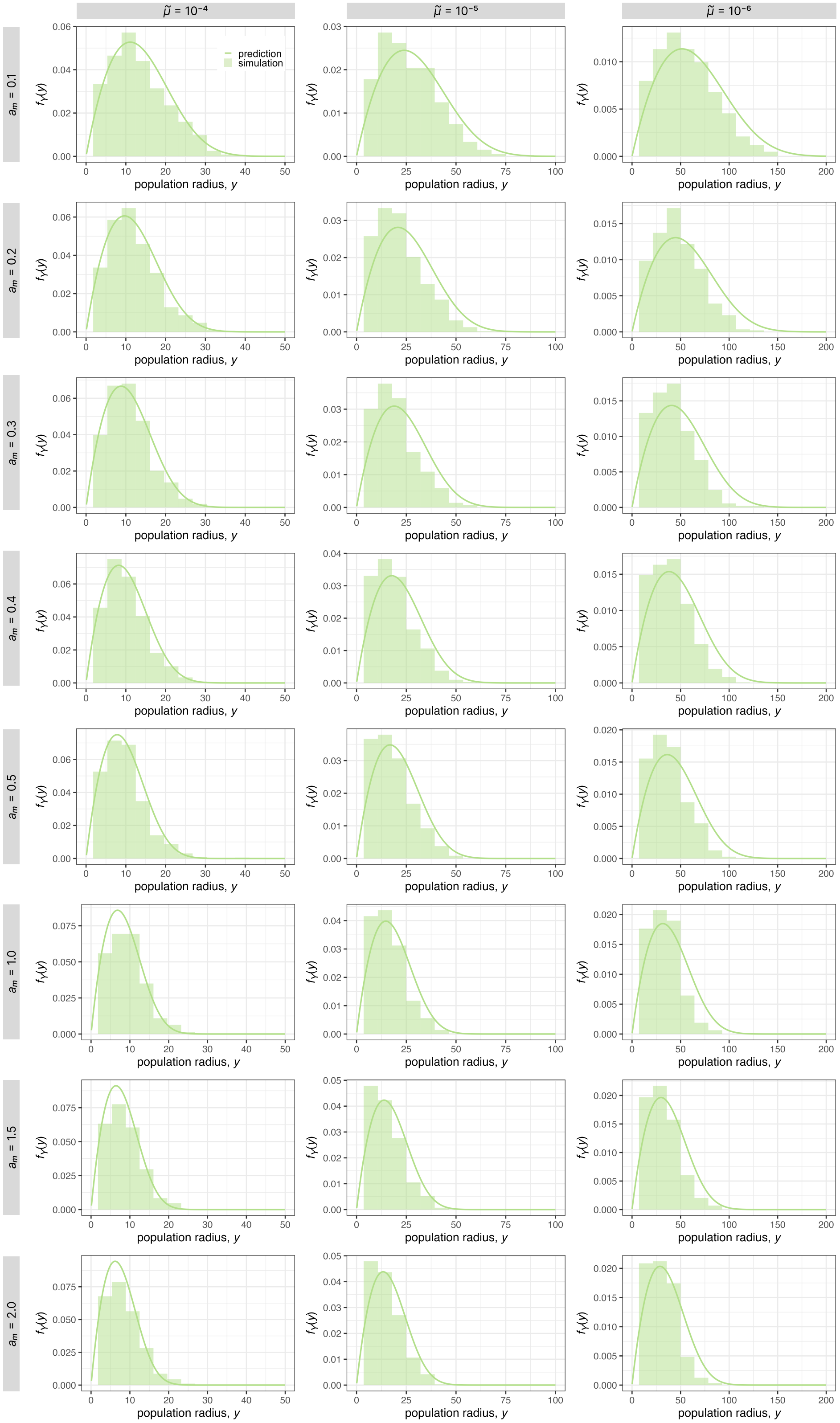

Fig. S3. Comparison of simulation results with our analytic predictions of f_Y in 2D for different parameter combinations. We use $m = 0.05$, $K = 16$, $r_{\mathrm{re}} = 0.91$, $r_{\mathrm{wt}} = 1.00$. We vary over μ and r_{m} corresponding to different a_{m}. Conversion between simulation and macroscopic model parameters is described in the Materials and Methods.

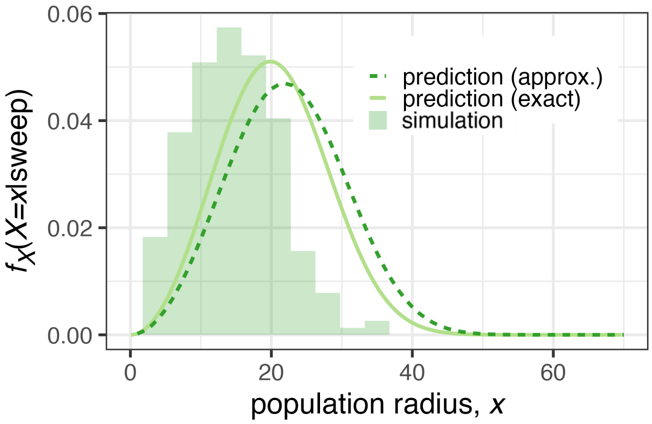

Fig. S4. Comparison of simulation results with our analytic predictions of $f_X(x|\text{sweep})$ in 2D. We use $m = 0.05$, $K = 16$, $r_{\text{re}} = 0.91$, $r_{\text{wt}} = 1.00$, $r_{\text{m}} = 1.3$ and $\tilde{\mu} = 10^{-5}$ leading to speeds $c_{\text{wt}} = 0.15$ and $c_{\text{m}} = 0.31$ and survival probability $\rho = 0.23$ (see Materials and Methods).

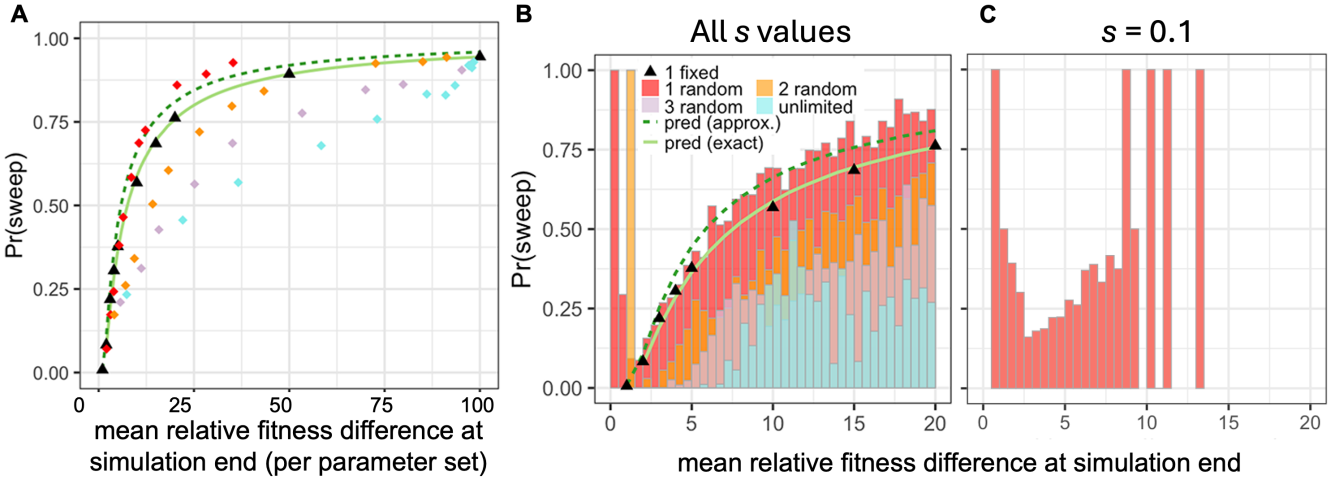

Fig. S5. Sweep probability versus mean relative fitness difference at simulation end. A sweep is counted irrespective of whether it was achieved by the first, the second, or a subsequent arising mutation. The x-axis is the mean value of $a_x/a_{wt} = (r_x - r_{wt})/(r_{wt} - r_{re})$ at the end of simulation, where x is either m (for mutant individuals) or wt (wildtype individuals). See SI Text Section 9 for more details. **A.** In this case we first find the mean of a_x/a_{wt} across all mutant and wildtype individuals at the end of each simulation and we then calculate the mean of these simulation means for each batch of 1,000 simulations with identical parameter values. Each black point is based on 1,000 replicates of the model with equal mutation effects. Each coloured point is each based on 1,000 replicates of a model that allows individuals to accumulate up to one (red), two (orange), three (purple) or an unlimited number (blue) of mutations with random fitness effects. Green curves represent approximate (dashed curve) and exact (solid curve) analytical predictions. **B.** Here we instead pool the simulation means from all values of parameter s and assign them to bins. **C.** A plot of the same kind as B but including results of only one batch of simulations ($s = 0.1$) of the model that allows individuals to accumulate up to one mutation. Fixed parameter values for all three plots are $m = 0.05$, $\mu = 10^{-5}$, $K = 16$, $r_{re} = 0.91$ and $r_{wt} = 1$.

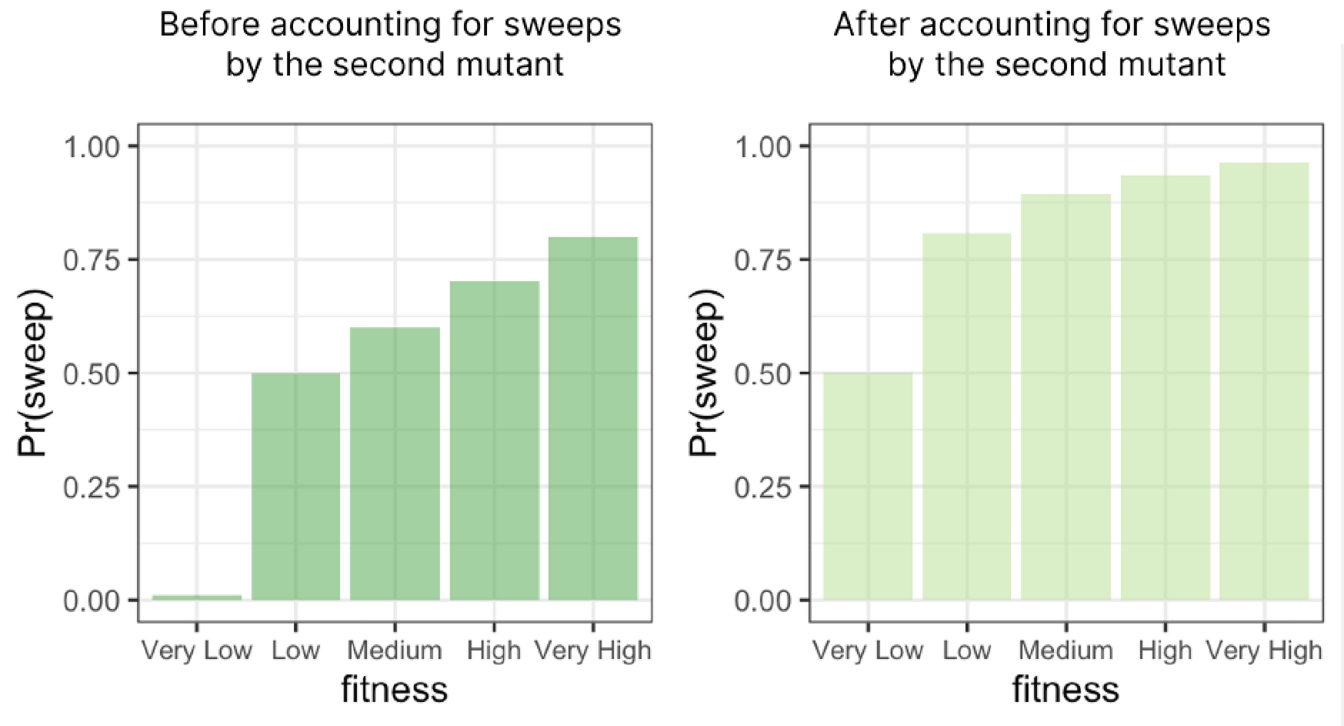

Fig. S6. Illustration of why, in the random-mutation-effects model, the sweep frequency has a peak associated with very low final fitness. The first plot shows sweep frequencies associated with five fitness values when we assume that the outcome of two competing mutants must be a non-sweep. The second plot shows the sweep frequencies after accounting for sweeps in cases where the second mutant is fitter than the first. The distribution of fitness effects is (0.01, 0.2475, 0.2475, 0.2475, 0.2475) and the sweep probabilities associated with the five fitness values are 0.01, 0.5, 0.6, 0.7 and 0.8, respectively.

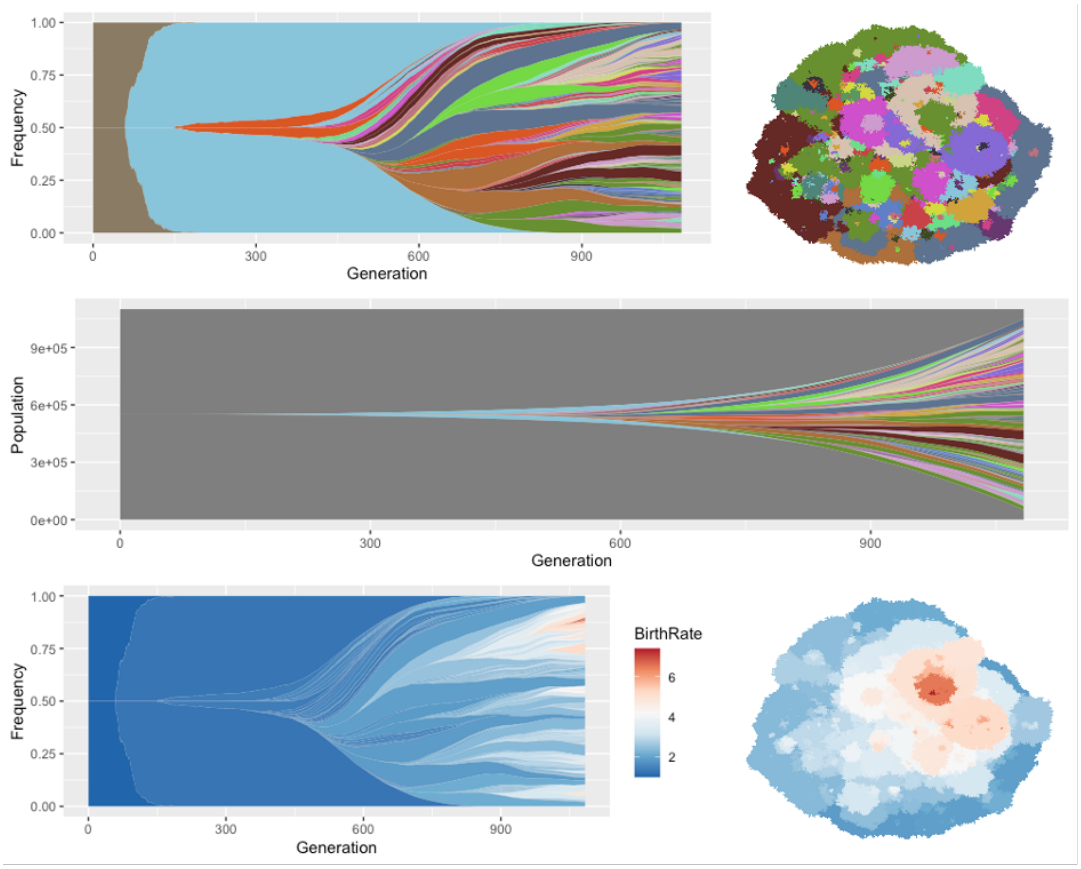

Fig. S7. Example of a selective sweep in a two-dimensional agent-based simulation in which mutations have multiplicative random effects on fitness and the accumulation of mutations is unrestricted. In the top three plots, distinct mutants are represented by different colours. In the bottom two plots, mutants are coloured according to their relative fitness. Input parameters were set at $m = 0.05$, $\mu = 10^{-5}$, $K = 16$, $r_{re} = 0.91$, $r_{wt} = 1$ and $r_m = 1.1$

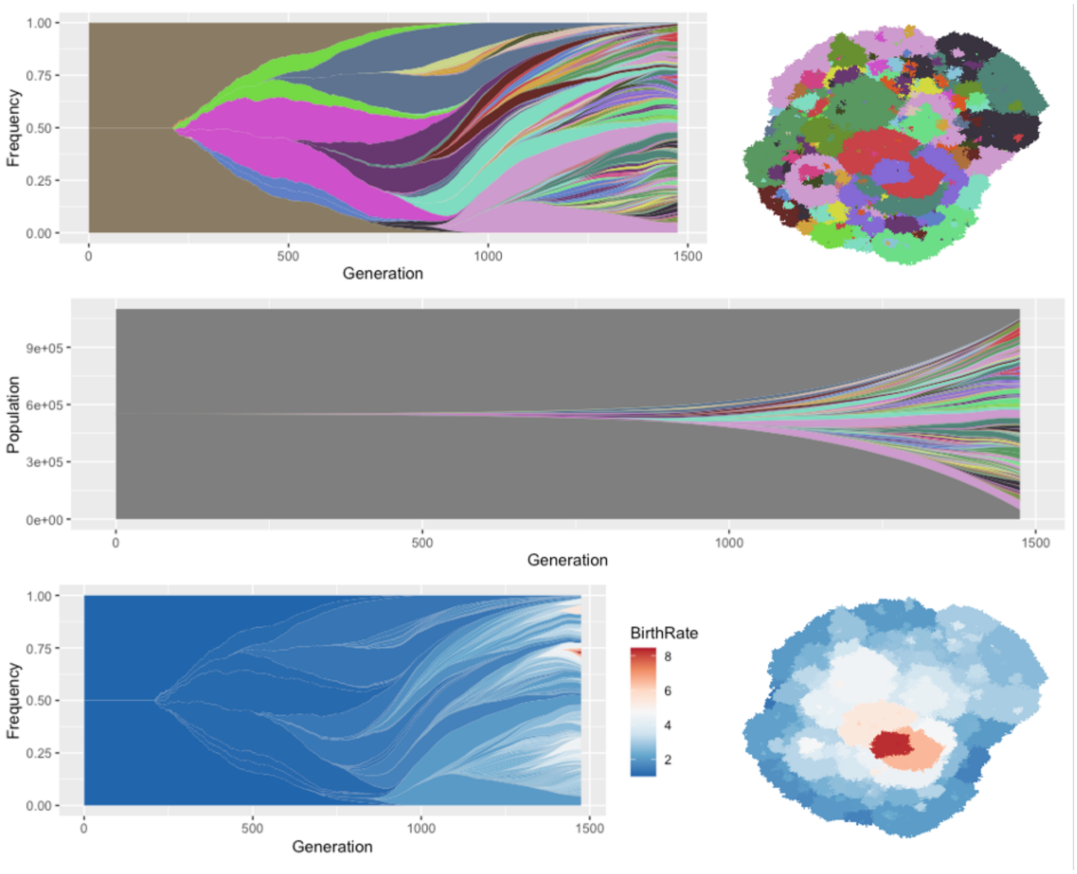

Fig. S8. Example of clonal interference preventing a sweep in a two-dimensional agent-based simulation in which mutations have multiplicative random effects on fitness and the accumulation of mutations is unrestricted. In the top three plots, distinct mutants are represented by different colours. In the bottom two plots, mutants are coloured according to their relative fitness. Input parameters were set at $m = 0.05$, $\mu = 10^{-5}$, $K = 16$, $r_{re} = 0.91$, $r_{wt} = 1$ and $r_m = 1.1$

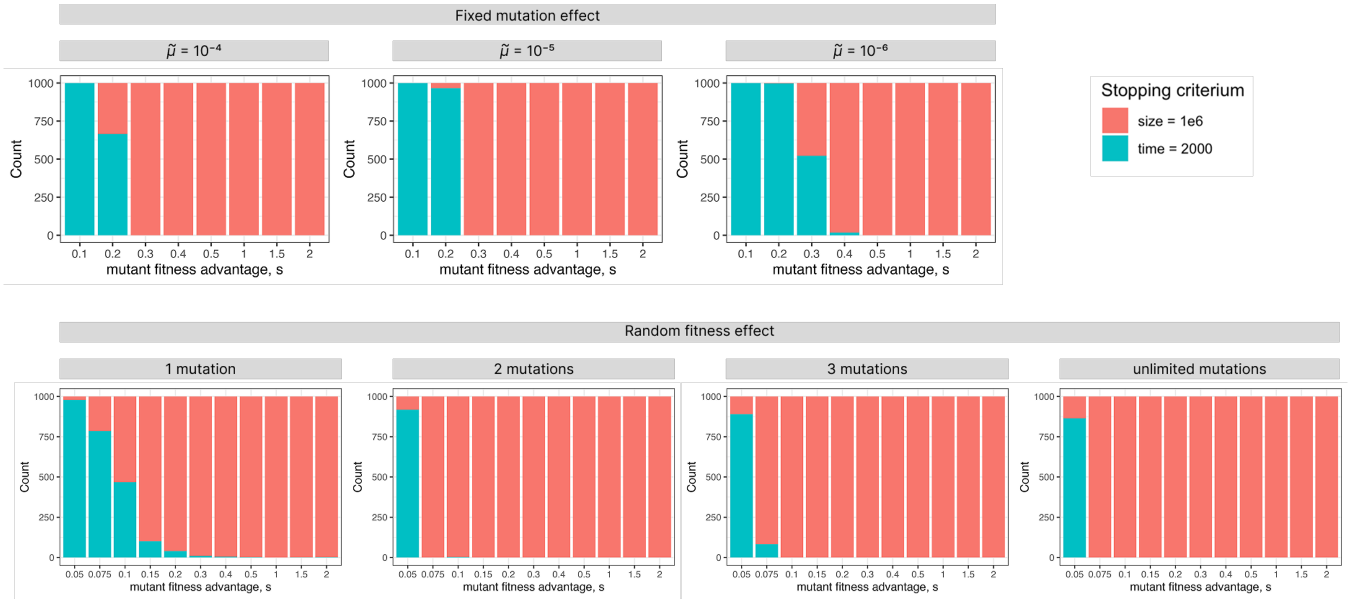

Fig. S9. Proportion of simulations stopped because of reaching the maximum size (10^6 individuals) or maximum time (2,000 generations) in simulations with equal mutation effects (top row) and random fitness effects (bottom row).

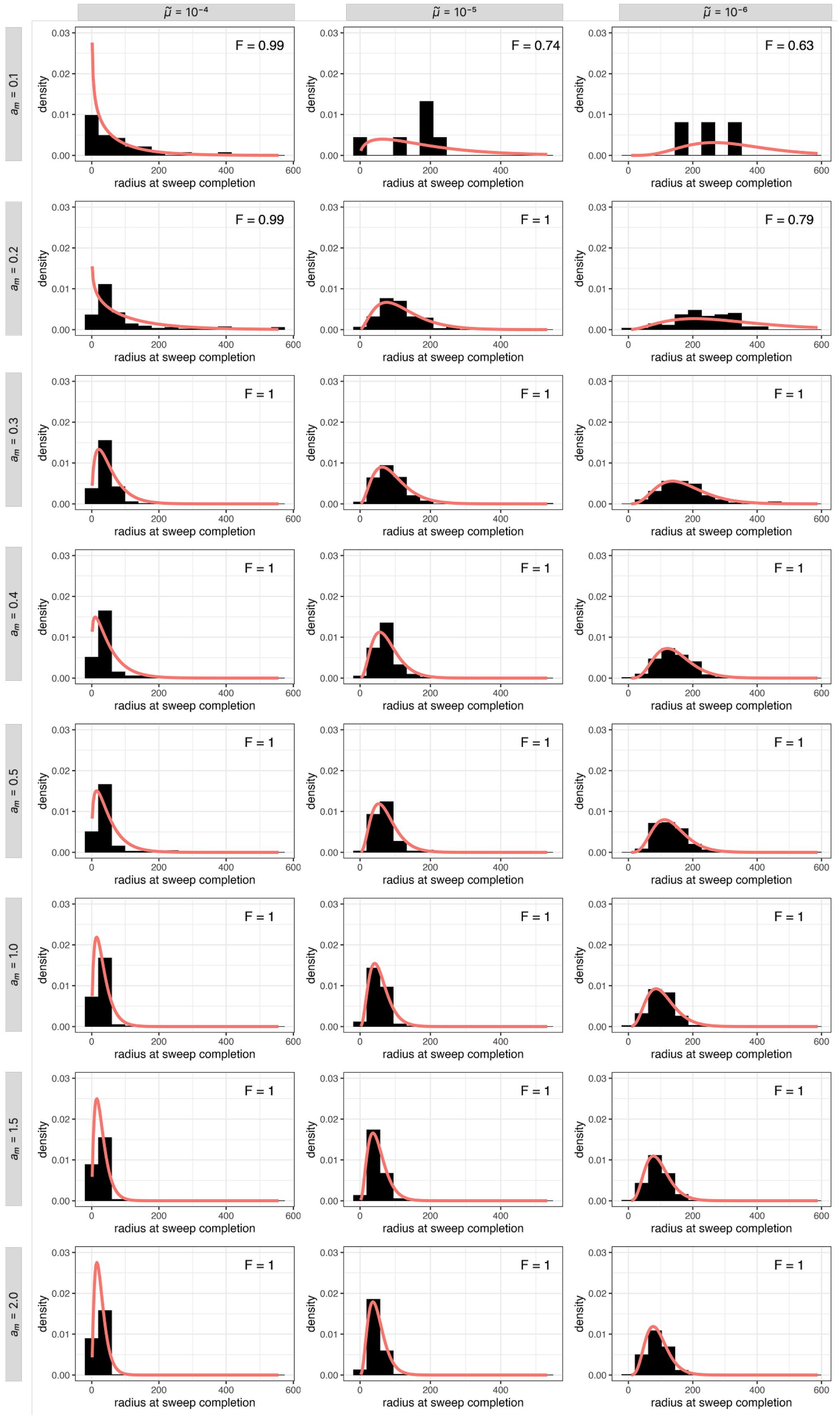

Fig. S10. Sweep completion radii for simulations with fixed mutation effects. Histograms show the radii at which the sweep completed, given that a sweep occurred in the simulation. Orange lines show fitted gamma distribution. The F value is the inferred value of the fitted cumulative distribution function at the effective maximum radius $F = F(r_{\text{max}} = 564)$, which estimates the percentage of sweeps captured.

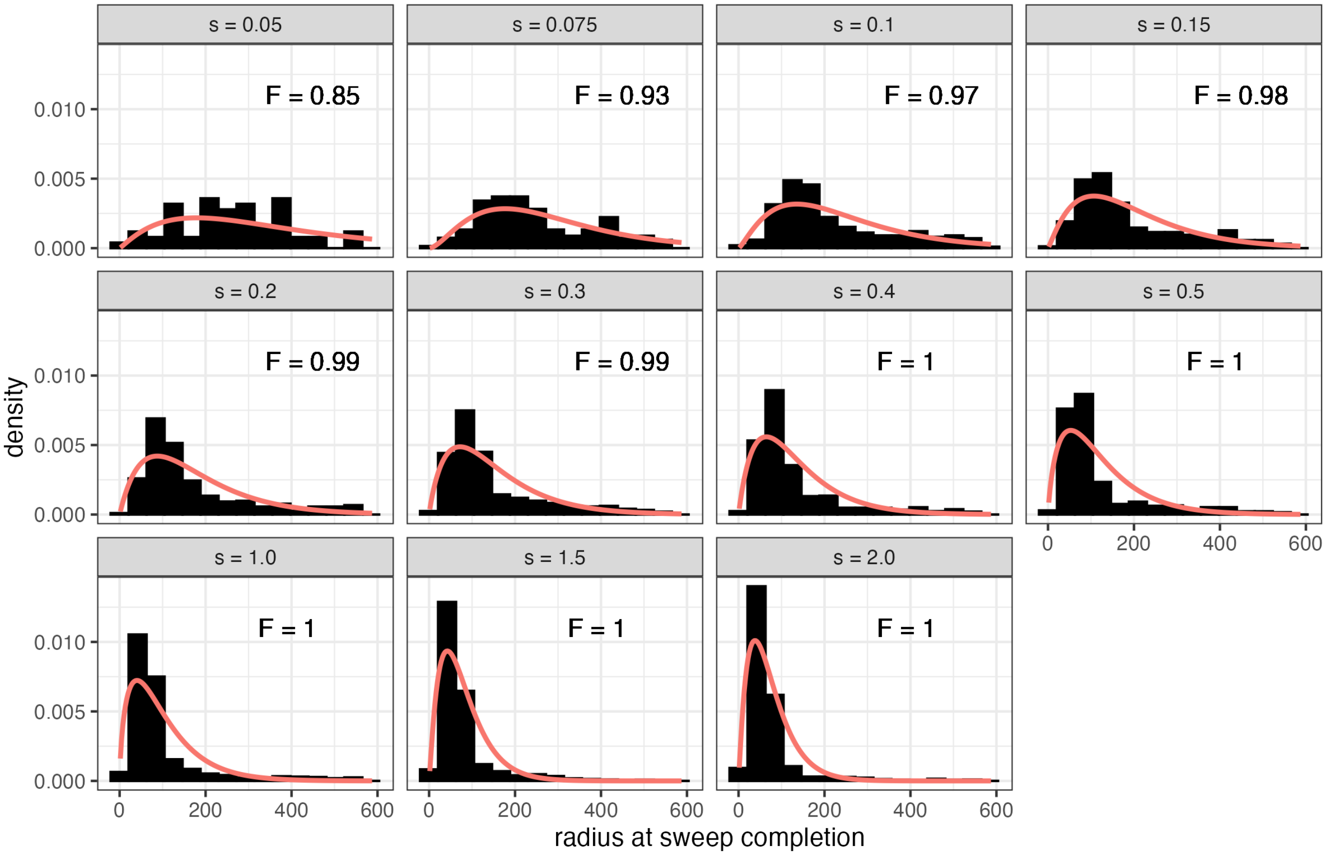

Fig. S11. Sweep completion radii in the case of maximum 1 mutation with random mutation effects. Histograms show the radii at which the sweep completed, given that a sweep occurred in the simulation. Orange lines show fitted right-truncated gamma distribution. The F value is the inferred value of the fitted cumulative distribution function at the effective maximum radius $F = F(r_{\mathrm{max}} = 564)$, which estimates the percentage of sweeps captured.

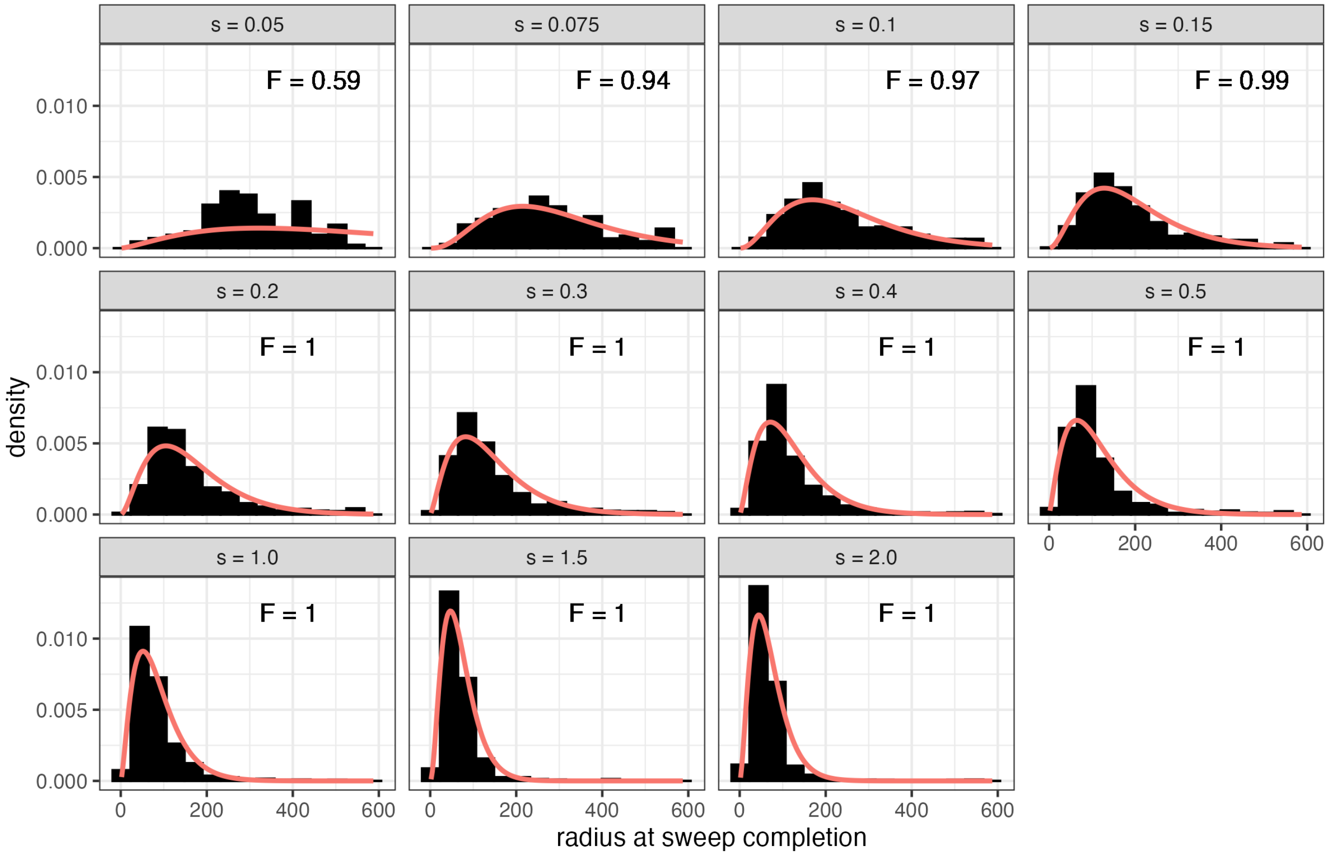

Fig. S12. Sweep completion radii in the case of maximum 2 mutations with random mutation effects. Histograms show the radii at which the sweep completed, given that a sweep occurred in the simulation. Orange lines show fitted right-truncated gamma distribution. The F value is the inferred value of the fitted cumulative distribution function at the effective maximum radius $F = F(r_{\text{max}} = 564)$, which estimates the percentage of sweeps captured.

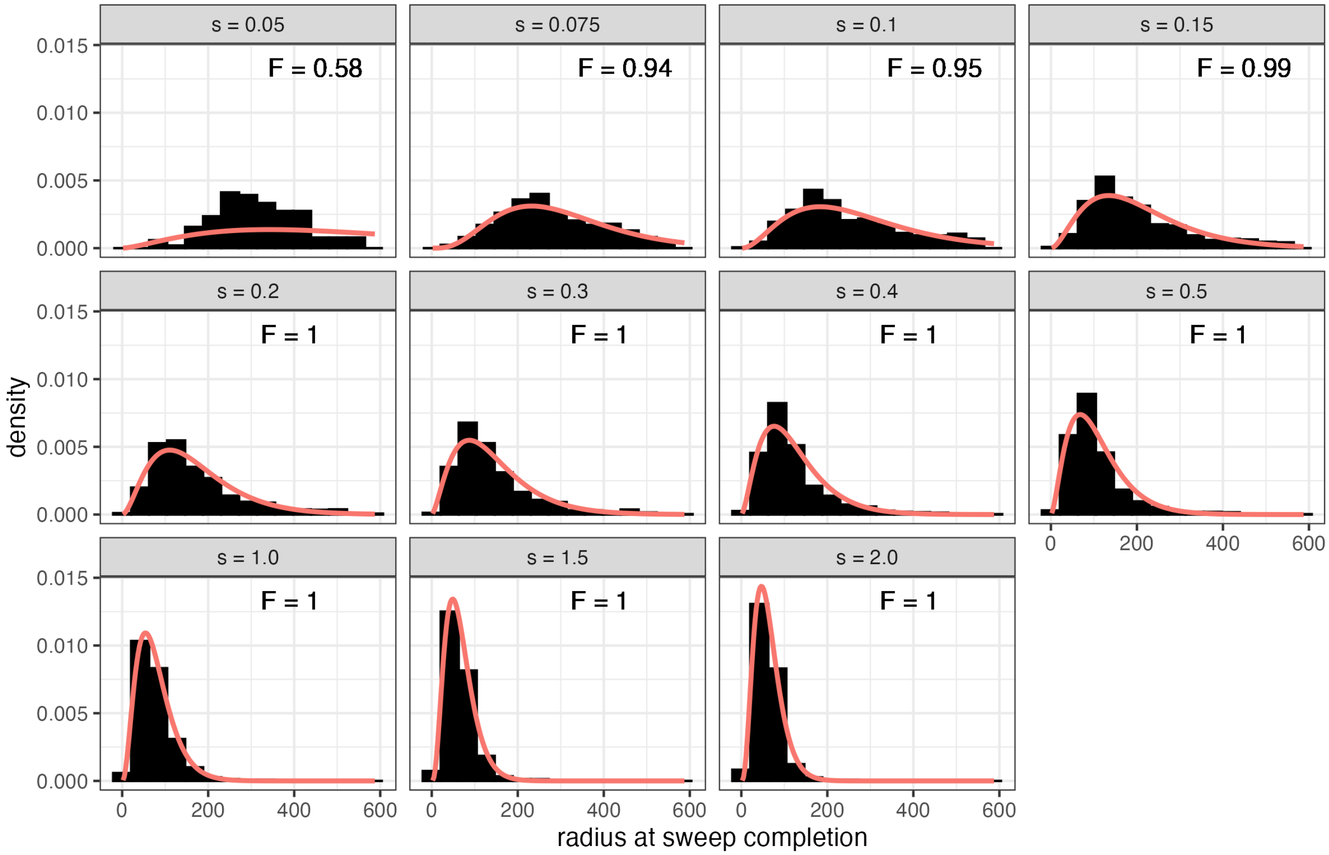

Fig. S13. Sweep completion radii in the case of maximum 3 mutations with random mutation effects. Histograms show the radii at which the sweep completed, given that a sweep occurred in the simulation. Orange lines show fitted right-truncated gamma distribution. The F value is the inferred value of the fitted cumulative distribution function at the effective maximum radius $F = F(r_{\text{max}} = 564)$, which estimates the percentage of sweeps captured.

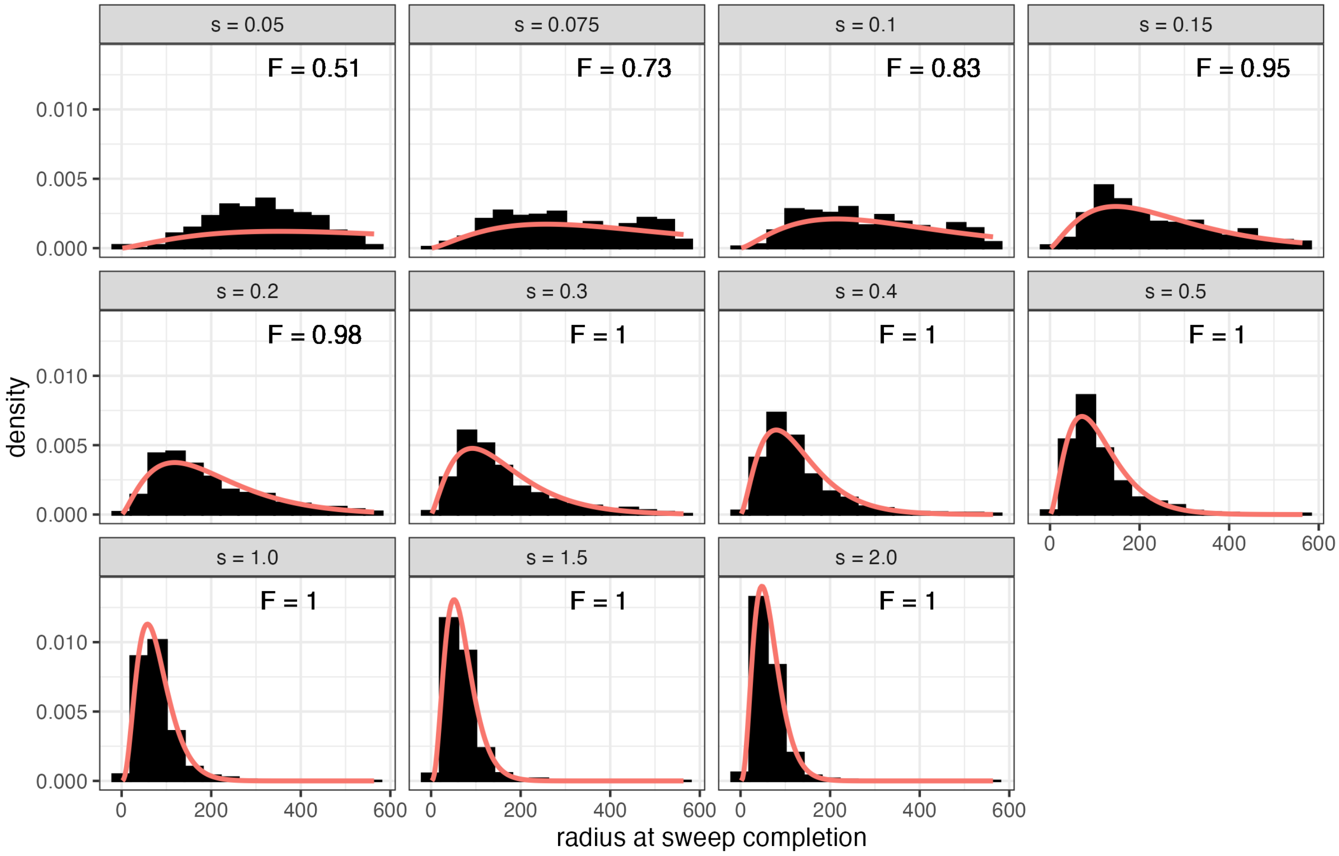

Fig. S14. Sweep completion radii in the case of unlimited mutations with random mutation effects. Histograms show the radii at which the sweep completed, given that a sweep occurred in the simulation. Orange lines show fitted right-truncated gamma distribution. The F value is the inferred value of the fitted cumulative distribution function at the effective maximum radius $F = F(r_{\text{max}} = 564)$, which estimates the percentage of sweeps captured.

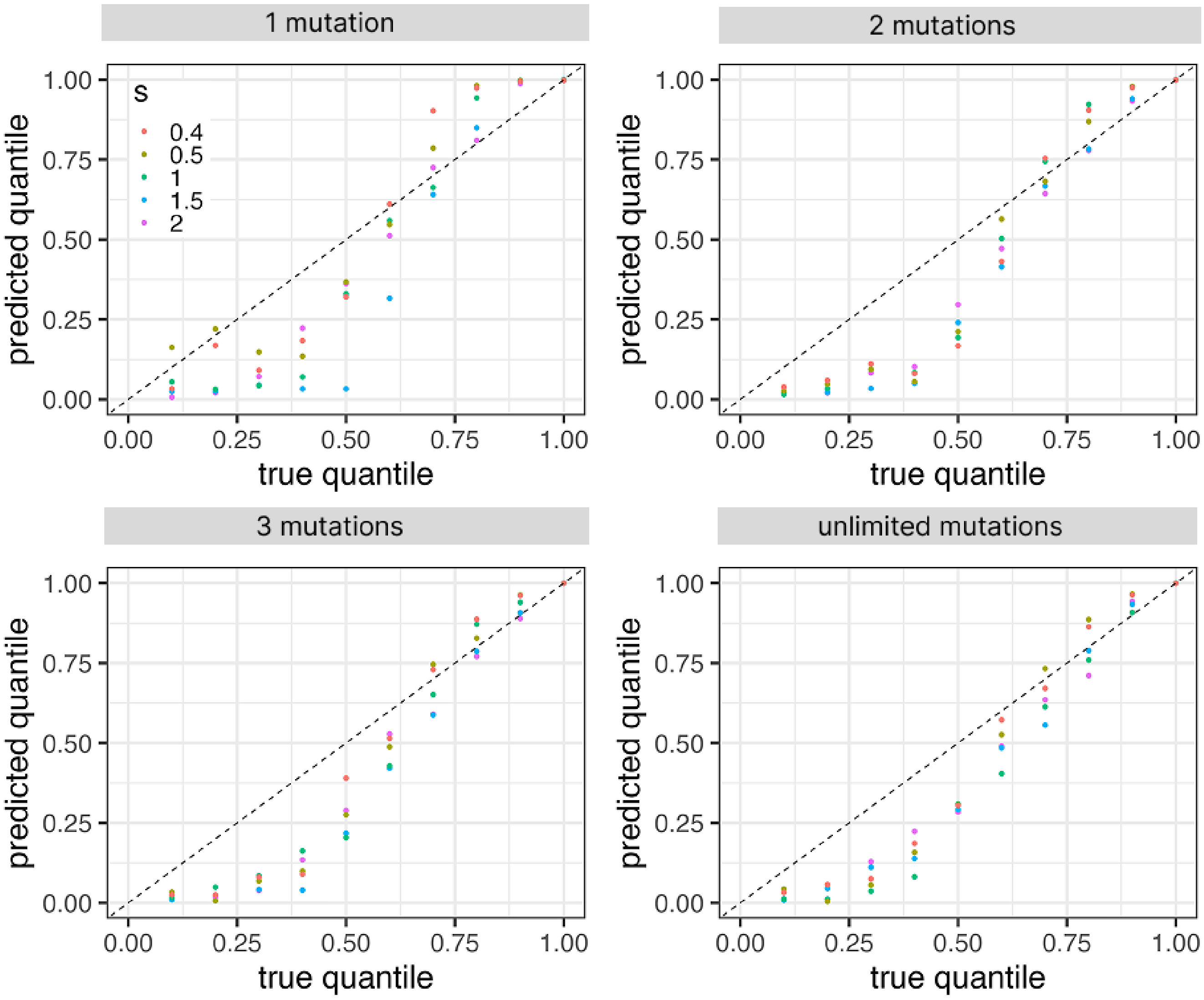

Fig. S15. Assessment of how well our statistical method infers the extent of undercounting of sweeps. For simulations that we assume captured the vast majority of sweeps ($s \geq 0.4$), we artificially truncated the radius distributions at quantiles 0.1, 0.2, 0.3, 0.4, 0.5, 0.6, 0.7, 0.8, 0.9 and 1.0 (x axis). We then fitted a truncated gamma distribution to infer the truncation point (y axis). Points above the diagonal correspond to underestimation of the extent of missing data; points below the diagonal correspond to overestimation.

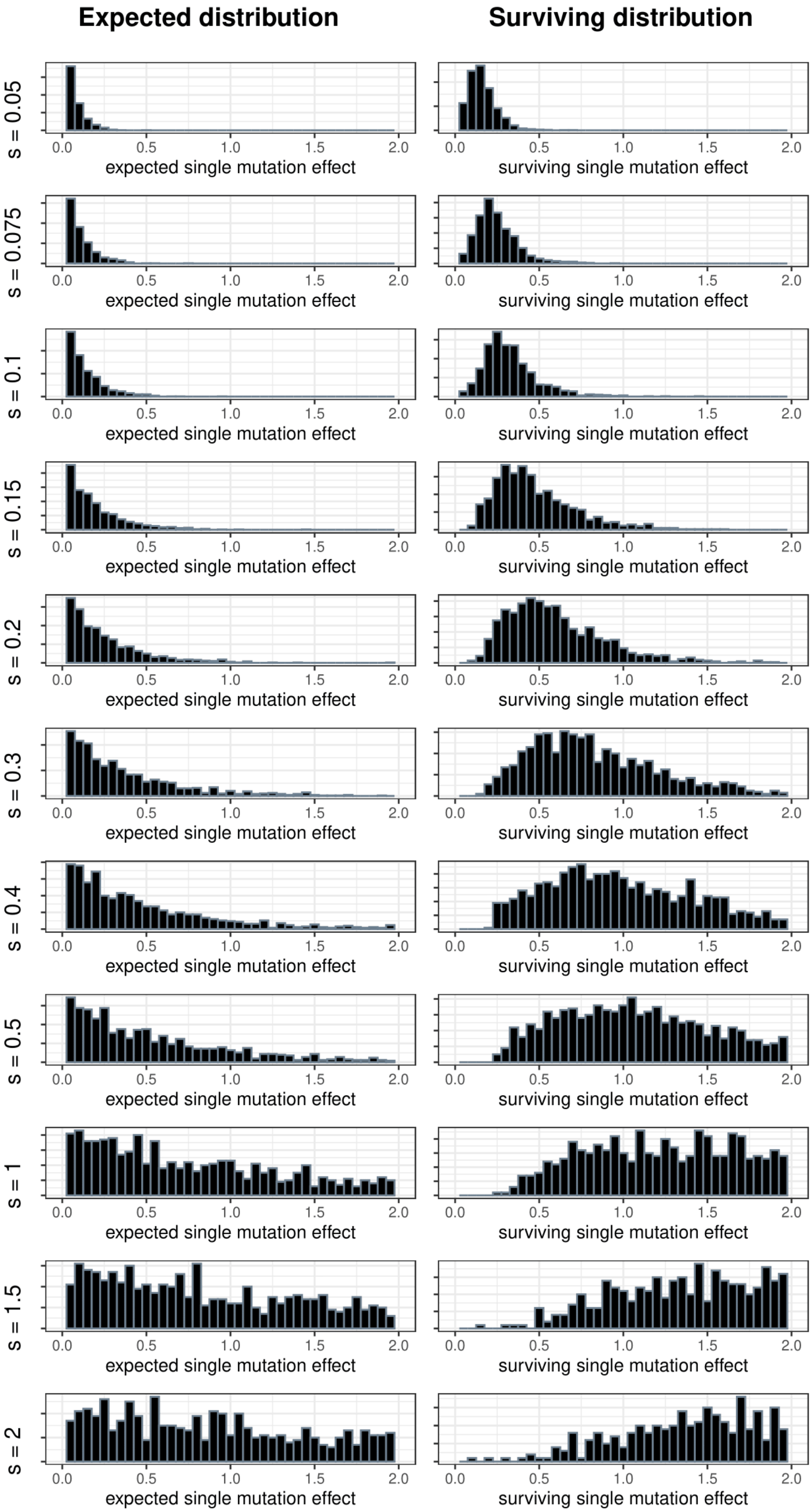

Fig. S16. Mutation effect histograms for all generated mutations (left column) and contending mutations that evaded stochastic extinction (right column).

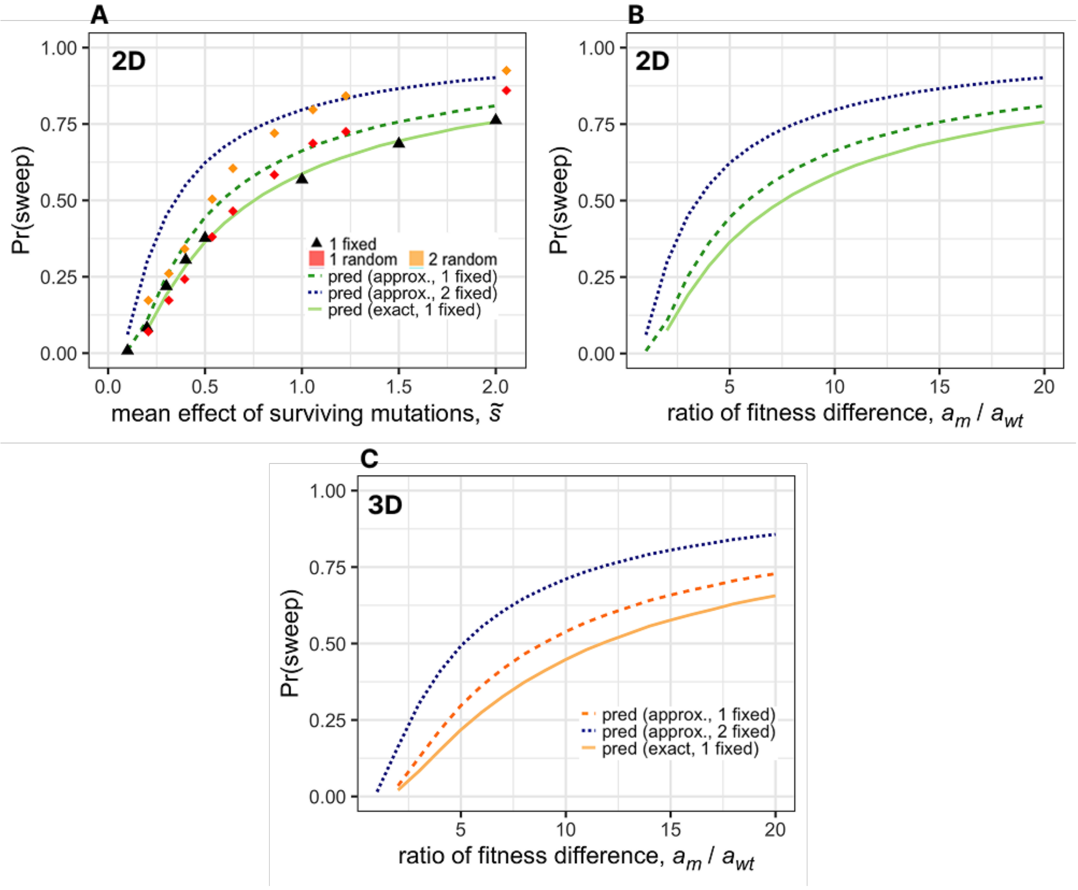

Fig. S17. Upper bound approximation for selective sweep probabilities when individuals can acquire two fitness-enhancing mutations given by eqn. 78 (displayed here as dotted dark blue line). **A.** Comparison with simulation results in 2D plotted against mean effect of surviving mutations. **B.** Comparison with analytic results in 2D with maximum 1 mutation. **C.** Comparison with analytic results in 3D with maximum 1 mutation. We assume that the relationship of speed ratios to fitness ratios $\frac{c_{\mathrm{wt}}}{c_{\mathrm{m}}} \sim \frac{s_{[wt]}}{s_{[m]}}$ is in 3D the same as in 2D.

	1D	2D	3D
$f_X(x)$	$\frac{2x}{\theta_{1D}^2} e^{-\frac{x^2}{\theta_{1D}^2}}$	$\frac{3x^2}{\theta_{2D}^3} e^{-\frac{x^3}{\theta_{2D}^3}}$	$\frac{4x^3}{\theta^4} e^{-\frac{x^4}{\theta^4}}$
$f_Y(y\|X=x)$	$\frac{1}{x} 1\{y \leq x\}$	$\frac{2y}{x^2} 1\{y \leq x\}$	$\frac{3y^2}{x^3} 1\{y \leq x\}$
$f_Y(y)$	$\frac{1}{\theta_{1D}} \Gamma\left(\frac{1}{2}, \frac{y^2}{\theta_{1D}^2}\right)$	$\frac{2y}{\theta_{2D}^2} \Gamma\left(\frac{1}{3}, \frac{y^3}{\theta_{2D}^3}\right)$	$\frac{3y^2}{\theta^3} \Gamma\left(\frac{1}{4}, \frac{y^4}{\theta^4}\right)$
Pr(sweep$\|X=x, Y=y$)	$e^{-\frac{x^2+y^2}{\alpha_{1D}^2}}$	numerical evaluation	$e^{-\mu\left(Ax^4+By^4+Cx^2y^2\right)}$
$\Pr($sweep$\|X=x)$	$\frac{\sqrt{\pi}\alpha_{1D}}{2x} e^{-\left(\frac{x}{\alpha_{1D}}\right)^2}$ erf $\left(\frac{x}{\alpha_{1D}}\right)$	numerical evaluation	numerical evaluation
Pr(sweep$\|X=x, Y=0$)	$e^{-\left(\frac{x}{\alpha_{1D}}\right)^2}$	$e^{-\left(\frac{x}{\alpha_{2D}}\right)^3}$	$e^{-\left(\frac{x}{\alpha}\right)^4}$
Pr(sweep) [approx.]	$\frac{c_{\text{m}}-c_{\text{wt}}}{c_{\text{m}}}$	$\left(\frac{c_{\text{m}}-c_{\text{wt}}}{c_{\text{m}}}\right)^2$	$\left(\frac{c_{\text{m}}-c_{\text{wt}}}{c_{\text{m}}}\right)^3$
Pr(sweep) [exact]	$\frac{\beta'}{\sqrt{1+\beta'}} \cot^{-1}\left(\sqrt{1+\beta'}\right)$	numerical evaluation	numerical evaluation
$f_X(X=x\|$sweep) [approx.]	$\frac{2x}{\theta_{1D}^2\beta} e^{-\frac{x^2}{\theta_{1D}^2\beta}}$	$\frac{3x^2}{\theta_{2D}^3\beta^2} e^{-\frac{x^3}{\theta_{2D}^3\beta^2}}$	$\frac{4x^3}{\theta^4\beta^3} e^{-\frac{x^4}{\theta^4\beta^3}}$
$f_X(X=x\|$sweep) [exact]	$\frac{\Pr(\text{sweep}\|X=x)f_X(x)}{\Pr(\text{sweep})}$	numerical evaluation	numerical evaluation

Table S1. Summary of analytical results of our main model for 1D, 2D and 3D.

Model	Sweep probability	Reference
boundary growth and cell proliferation throughout the population	$\int_0^\infty \int_0^x e^{-\mu \int_0^\infty N_{\text{wt}}(\tau)\, d\tau} \frac{3y^2}{x^3} \frac{4x^3 e^{-x^4/\theta^4}}{\theta^4}\, dy\, dx$ $\leq \left(\frac{c_{\text{m}} - c_{\text{wt}}}{c_{\text{m}}}\right)^3$	here
boundary growth and cell proliferation restricted to the boundary	$\frac{8+\frac{c_{\text{m}}}{c_{\text{wt}}}}{\frac{c_{\text{m}}}{c_{\text{wt}}}-1} \frac{2}{1+\exp\left(3\pi/(\frac{c_{\text{m}}}{c_{\text{wt}}}-1)\right)}$	Ref. (8)
constant population with radially growing mutant	$e^{-\frac{\mu\pi x_0^4}{c_{\text{m}}}}$	Ref. (4, 5) and here
exponential growth	$\int_0^\infty e^{-\frac{\mu}{r_{\text{wt}}} e^{rt_1}\left(e^{r_{\text{wt}} t_2(t_1)}-1\right)}$ $\times \mu e^{rt_1} e^{-\frac{\mu}{r} e^{rt_1}}\, dt_1$	here
constant population with logistically growing mutant	$N_0 e^{-\mu \frac{N_0}{s}}$	Ref. (10) and here

Table S2. Summary of sweep probabilities for different growth models in 3D.

a_m	0.1	0.2	0.3	0.4	0.5	0.6	0.7
c_m	0.14	0.23	0.31	0.38	0.46	0.53	0.60

a_m	0.8	0.9	1.0	1.1	1.2	1.3	1.4
c_m	0.68	0.75	0.82	0.89	0.96	1.03	1.11

a_m	1.5	1.6	1.7	1.8	1.9	2.0	
c_m	1.17	1.24	1.31	1.39	1.46	1.52	

Table S3. Correspondence between difference in proliferation rates a_m and mutant propagation speed c_m, measured in simulations.

References

1. EW Weisstein, Sphere-sphere intersection. from mathworld–a wolfram web resource. (https://mathworld.wolfram.com/Sphere-SphereIntersection.html) (2007) Accessed: 22/11/2023.
2. R Lyons, Another contender in the arctangent race. *IEEE Signal Process. Mag.* **21**, 109–110 (2004).
3. EW Weisstein, Circle-circle intersection. from mathworld–a wolfram web resource. (https://mathworld.wolfram.com/Circle-CircleIntersection.html) (2003) Accessed: 22/11/2023.
4. P Ralph, G Coop, Parallel adaptation: one or many waves of advance of an advantageous allele? *Genetics* **186**, 647–668 (2010).
5. EA Martens, O Hallatschek, Interfering waves of adaptation promote spatial mixing. *Genetics* **189**, 1045–1060 (2011).
6. EA Martens, R Kostadinov, CC Maley, O Hallatschek, Spatial structure increases the waiting time for cancer. *New journal physics* **13**, 115014 (2011).
7. R Durrett, *Branching process models of cancer*, Mathematical Biosciences Institute Lecture Series. (Springer), (2015).
8. T Antal, P Krapivsky, M Nowak, Spatial evolution of tumors with successive driver mutations. *Phys. Rev. E* **92**, 022705 (2015).
9. KS Korolev, et al., Selective sweeps in growing microbial colonies. *Phys. biology* **9**, 026008 (2012).
10. PJ Gerrish, RE Lenski, The fate of competing beneficial mutations in an asexual population. *Genetica* **102**, 127–144 (1998).
11. S Benzekry, et al., Classical mathematical models for description and prediction of experimental tumor growth. *PLoS computational biology* **10**, e1003800 (2014).
12. W Huang, C Hauert, A Traulsen, Stochastic game dynamics under demographic fluctuations. *Proc. Natl. Acad. Sci.* **112**, 9064–9069 (2015).
